# A seventeenth-century *Mycobacterium tuberculosis* genome supports a Neolithic emergence of the *Mycobacterium tuberculosis* complex

**DOI:** 10.1186/s13059-020-02112-1

**Published:** 2020-08-10

**Authors:** Susanna Sabin, Alexander Herbig, Åshild J. Vågene, Torbjörn Ahlström, Gracijela Bozovic, Caroline Arcini, Denise Kühnert, Kirsten I. Bos

**Affiliations:** 1 Department of Archaeogenetics, Max Planck Institute for the Science of Human History, 07745 Jena, Germany; 2 Present address: Section for Evolutionary Genomics, The GLOBE Institute, University of Copenhagen, 1353 Copenhagen, Denmark; 3 Department of Archaeology and Ancient History, Lund University, 221 00 Lund, Sweden; 4 Department of Medical Imaging and Clinical Physiology, Skåne University Hospital Lund and Lund University, 221 00 Lund, Sweden; 5 Arkeologerna, National Historical Museum, 226 60 Lund, Sweden; 6 Transmission, Infection, Diversification & Evolution Group, Max Planck Institute for the Science of Human History, 07745 Jena, Germany

**Keywords:** Tuberculosis, Ancient DNA, *Mycobacterium tuberculosis*, Molecular dating, Metagenomics

## Abstract

**Background:**

Although tuberculosis accounts for the highest mortality from a bacterial infection on a global scale, questions persist regarding its origin. One hypothesis based on modern *Mycobacterium tuberculosis* complex (MTBC) genomes suggests their most recent common ancestor followed human migrations out of Africa approximately 70,000 years before present. However, studies using ancient genomes as calibration points have yielded much younger dates of less than 6000 years. Here, we aim to address this discrepancy through the analysis of the highest-coverage and highest-quality ancient MTBC genome available to date, reconstructed from a calcified lung nodule of Bishop Peder Winstrup of Lund (b. 1605–d. 1679).

**Results:**

A metagenomic approach for taxonomic classification of whole DNA content permitted the identification of abundant DNA belonging to the human host and the MTBC, with few non-TB bacterial taxa comprising the background. Genomic enrichment enabled the reconstruction of a 141-fold coverage *M*. *tuberculosis* genome. In utilizing this high-quality, high-coverage seventeenth-century genome as a calibration point for dating the MTBC, we employed multiple Bayesian tree models, including birth-death models, which allowed us to model pathogen population dynamics and data sampling strategies more realistically than those based on the coalescent.

**Conclusions:**

The results of our metagenomic analysis demonstrate the unique preservation environment calcified nodules provide for DNA. Importantly, we estimate a most recent common ancestor date for the MTBC of between 2190 and 4501 before present and for Lineage 4 of between 929 and 2084 before present using multiple models, confirming a Neolithic emergence for the MTBC.

## Background

Tuberculosis, caused by organisms in the *Mycobacterium tuberculosis* complex (MTBC), has taken on renewed relevance and urgency in the twenty-first century due to its global distribution, its high morbidity, and the rise of antibiotic-resistant strains [1]. The difficulty in disease management and treatment, combined with the massive reservoir the pathogen maintains in human populations through latent infection [2], makes tuberculosis a pressing public health challenge. Despite this, controversy exists regarding the history of the relationship between members of the MTBC and their human hosts.

Existing literature suggests two estimates for the time to the most recent common ancestor (tMRCA) for the MTBC based on the application of Bayesian molecular dating to genome-wide *Mycobacterium tuberculosis* data. One estimate suggests the extant MTBC emerged through a bottleneck approximately 70,000 years ago, coincident with major migrations of humans out of Africa [3]. This estimate was reached using a large global dataset of exclusively modern *M*. *tuberculosis* genomes, with internal nodes of the MTBC calibrated by extrapolated dates for major human migrations [3]. This estimate relied on congruence between the topology of the MTBC and human mitochondrial phylogenies, but this congruence does not extend to human Y chromosome phylogeographic structure [4]. As an alternative approach, the first publication of ancient MTBC genomes utilized radiocarbon dates as direct calibration points to infer mutation rates and yielded an MRCA date for the complex of less than 6000 years [5]. This younger emergence was later supported by mutation rates estimated within the pervasive Lineage 4 (L4) of the MTBC, using four *M*. *tuberculosis* genomes from the late eighteenth and early nineteenth centuries [6].

Despite the agreement in studies that have relied on ancient DNA calibration so far, dating of the MTBC emergence remains controversial. The young age suggested by these works cannot account for purported detection of MTBC DNA in archeological material that predates the tMRCA estimate (e.g., Baker et al. [7]; Hershkovitz et al. [8]; Masson et al. [9]; Rothschild et al. [10]), the authenticity of which has been challenged [11]. Furthermore, constancy in mutation rates of the MTBC has been questioned on account of observed rate variation in modern lineages, combined with the unquantified effects of latency [12]. The ancient genomes presented by Bos and colleagues, though isolated from human remains, were most closely related to *Mycobacterium pinnipedii*, a lineage of the MTBC currently associated with infections in seals and sea lions [5]. Given our unfamiliarity with the demographic history of tuberculosis in sea mammal populations [13], identical substitution rates between the pinniped lineage and human-adapted lineages of the MTBC cannot be assumed. Additionally, estimates of genetic diversity in MTBC strains from archeological specimens can be difficult given their similarities to environmental mycobacterial DNA from the depositional context, which increase the risk of false positive genetic characterization [14]. Though the ancient genomes published by Kay and colleagues belonged to human-adapted lineages of the MTBC, and the confounding environmental signals were significantly reduced by their funerary context in crypts, two of the four genomes used for molecular dating were derived from mixed-strain infections [6]. By necessity, diversity derived in each genome would have to be ignored for them to be computationally distinguished [6]. Though ancient DNA is a valuable tool for answering the question of when the MTBC emerged, the available ancient data remains sparse and subject to case-by-case challenges.

Here, we offer a higher resolution temporal estimate for the emergence of the MTBC and L4 using multiple Bayesian models of varying complexity through the analysis of a high-coverage seventeenth-century *M*. *tuberculosis* genome extracted from a calcified lung nodule. Removed from naturally mummified remains, the nodule provided an excellent preservation environment for the pathogen, and exhibited minimal infiltration by exogenous bacteria. The nodule and surrounding lung tissue also showed exceptional preservation of host DNA, thus showing promise for this tissue type in ancient DNA investigations.

## Results

### Pathogen identification

**Fig. 1:**
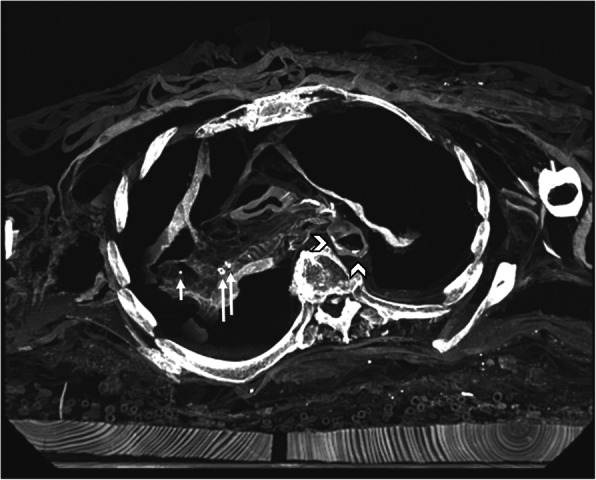
CT image of Ranke complex. CT image of Peder Winstrup’s chest in a slightly angled axial plane with the short arrow showing a small calcified granuloma in the probable upper lobe of the collapsed right lung, and two approximately 5 mm calcifications in the right hilum together suggesting a Ranke complex and previous primary tuberculosis. The more lateral of the two hilar calcifications was extracted for further analysis. In addition, there are calcifications in the descending aorta proposing atherosclerosis (arrowhead)

Computed tomography (CT) scans of the mummified remains of Bishop Peder Winstrup of Lund, Sweden revealed a calcified granuloma a few millimeters (mm) in size in the collapsed right lung together with two ~ 5 mm calcifications in the right hilum (Fig. 1). Primary tuberculosis causes parenchymal changes and ipsilateral hilar lymphadenopathy that is more common on the right side [15]. Upon resolution, it can leave a parenchymal scar, a small calcified granuloma (Ghon focus), and calcified hilar nodes, which are together called a Ranke complex. In imaging, this complex is suggestive of previous tuberculosis infection, although histoplasmosis can have the same appearance [16]. Histoplasmosis, however, is very rare in Scandinavia and is more often seen in other parts of the world (e.g., the Americas) [17]. The imaging findings were therefore considered to result from previous primary tuberculosis. One of the calcified hilar nodes was extracted from the remains during video-assisted thoracoscopic surgery, guided by fluoroscopy. The extracted material was further subsampled for genetic analysis. DNA was extracted from the nodule and accompanying lung tissue using protocols optimized for the recovery of ancient, chemically degraded, fragmentary genetic material [18]. The library (LUND1) was shotgun sequenced to a depth of approximately 3.7 million reads.

**Fig. 2:**
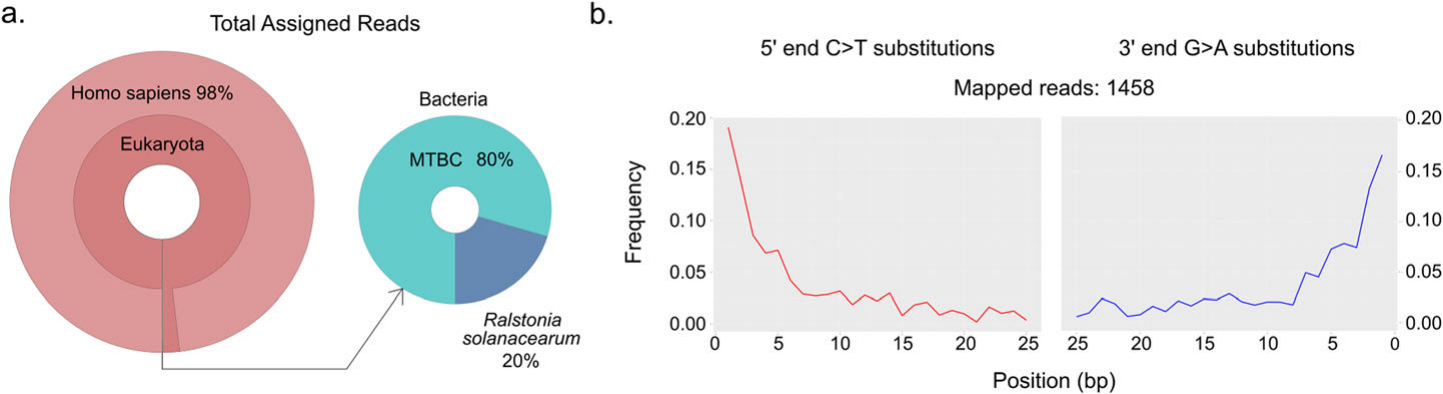
Screening of sequencing data from LUND1 shows preservation of host and pathogen DNA. **a** Krona plots reflecting the metagenomic composition of the lung nodule. The majority of sequencing reads were aligned to *Homo sapiens* (*n* = 2,833,403), demonstrating extensive preservation of host DNA. A small portion of reads aligned to bacterial organisms, and 80% of these reads were assigned to the MTBC node (*n* = 1724). **b** Damage plots generated from sequencing reads mapped directly to a reconstructed MTBC ancestor genome [21], demonstrating a pattern characteristic of ancient DNA

Adapter-clipped and base quality-filtered reads were taxonomically binned with MALT [19] against the full NCBI Nucleotide database (“nt,” April 2016). In this process, 3,515,715 reads, or 95% of the metagenomic reads, could be assigned to taxa contained within the database. Visual analysis of the metagenomic profile in MEGAN6 [20] revealed the majority of these reads, 2,833,403 or 81%, were assigned to *Homo sapiens*. A further 1724 reads were assigned to the *Mycobacterium tuberculosis* complex (MTBC) node. Importantly, no other taxa in the genus *Mycobacterium* were identified, and the only other identified bacterial taxon was *Ralstonia solanacearum* (Fig. 2a), a soil-dwelling plant pathogen frequently identified in metagenomic profiles of archeological samples [22, 23] (Additional File 1).

Pre-processed reads were mapped to both the hg19 human reference genome and a reconstructed MTBC ancestor (TB ancestor) [21] using BWA as implemented in the Efficient Ancient Genome Reconstruction (EAGER) pipeline [24]. Reads aligned to hg19 with direct mapping constituted an impressive 88% of the total sequencing data (Additional File 2). Human mitochondrial contamination was extremely low, estimated at only 1–3% using Schmutzi [25] (Additional File 3). Reads were also mapped to the TB ancestor (Table 1). After map quality filtering and read de-duplication, 1458 reads, or 0.045% of the total sequencing data, aligned to the reference (Table 1) and exhibited cytosine-to-thymine damage patterns indicative of authentic ancient DNA (Fig. 2b) [26, 27]. Qualitative preservation of the tuberculosis DNA was slightly better than that of the human DNA, as damage was greater in the latter (Additional File 2). Laboratory-based contamination, as monitored by negative controls during the extraction and library preparation processes, could be ruled out as the source of this DNA (Additional File 4).

**Table 1:** Mapping statistics for LUND1 libraries

Pre/post capture	Library treatment	Processed reads pre-mapping (*n*)	Unique mapped reads, quality-filtered (*n*)	Endogenous DNA (%)	Mean fold coverage	Mean fragment length (bp)	GC content (%)
Pre-capture	Non-UDG	3,696,712	1458	0.045	0.018	54.31	63.89
Post-capture	UDG	59,091,507	9,482,901	45.652	141.5062	65.83	62.96

A comparison of the mapping statistics for the non-UDG screening library and UDG-treated MTBC enriched library of LUND1 when aligned to the MTBC ancestor genome [21]. For full EAGER output, see Additional File 2

### Genomic enrichment and reconstruction

Due to the clear but low-abundance MTBC signal, a uracil DNA glycosylase (UDG) library was constructed to remove DNA lesions caused by hydrolytic deamination of cytosine residues [28] and enriched with an in-solution capture [29, 30] designed to target genome-wide data representing the full diversity of the MTBC (see the “Methods” section). The capture probes are based on a reconstructed TB ancestor genome [21]. The enriched library was sequenced using a paired-end, 150-cycle Illumina sequencing kit to obtain a full fragment-length distribution (Fig. S1 in Additional File 3). The resulting sequencing data was then aligned to the hypothetical TB ancestor genome [21], and the mapping statistics were compared with those from the screening data to assess enrichment (Table 1). Enrichment increased the proportion of endogenous MTBC DNA content by three orders of magnitude, from 0.045 to 45.652%, and deep sequencing yielded genome-wide data at an average coverage of approximately 141.5-fold. The mapped reads have an average fragment length of ~ 66 base pairs (Table 1).

We further evaluated the quality of the reconstructed genome by quantifying the amount of heterozygous positions (see the “Methods” section). Derived alleles represented by 10–90% of the reads covering a given position with five or more reads of coverage were counted. Only 24 heterozygous sites were counted across all variant positions in LUND1. As a comparison, the other high-coverage (~ 125 fold) ancient genome included here—body92 from Kay et al. [6]—contained 70 heterozygous positions.

### Phylogeny and dating

Preliminary phylogenetic analysis using neighbor joining (Figs. S2 and S3 in Additional File 3), maximum likelihood (Figs. S4 and S5 in Additional File 3), and maximum parsimony trees (Figs. S6 and S7 in Additional File 3) indicated that LUND1 groups within the L4 strain diversity of the MTBC, and more specifically, within the L4.10/PGG3 sublineage. This sublineage was recently defined by Stucki and colleagues as the clade containing L4.7, L4.8, and L4.9 [31] according to the widely accepted Coll nomenclature [32]. Following this, we constructed two datasets to support molecular dating of the full MTBC (Additional File 5) and L4 of the MTBC (Additional File 6).

The dataset reflecting extant diversity of the MTBC was compiled as reported elsewhere [5], with six ancient genomes as calibration points. These included LUND1; two additional ancient genomes, body80 and body92, extracted from late 18th and early nineteenth century Hungarian mummies [6]; and three human-isolated *Mycobacterium pinnipedii* strains from Peru [5], encompassing all available ancient *M*. *tuberculosis* genomes with sufficient coverage to call SNPs confidently after stringent mapping with BWA [33] (see the “Methods” section; Additional File 5). *Mycobacterium canettii* was used as an outgroup. In generating an alignment of variant positions in this dataset, we excluded repetitive regions and regions at risk of cross-mapping with other organisms as done previously [5], as well as potentially imported sites from recombination events, which were identified using ClonalFrameML [34] (Additional File 7). We chose to exclude these potential recombinant sites despite *M*. *tuberculosis* being generally recognized as a largely clonal organism with no recombination or horizontal gene transfer, as these phenomena have been found to occur in *M*. *canettii* [35, 36]. Only twenty-three variant sites were lost from the full MTBC alignment as potential imports. We called a total of 42,856 variable positions in the dataset as aligned to the TB ancestor genome. After incompletely represented sites were excluded, 11,716 were carried forward for downstream analysis. Prior to performing the Bayesian molecular dating analysis, we assessed the dataset for clock-like structure with TempEst (*R*^2^ = 0.273; see the “Methods” section; Fig. S8 in Additional File 3).

To explore the impact of the selected tree prior and clock model, we ran multiple variations of models as available for use in BEAST2 [37]. We first used both a strict and a relaxed clock model together with a constant coalescent model (CC+strict, CC+UCLD). We found there to be minimal difference between the inferred rates estimated by the two models. This finding, in addition to the low rate variance estimated in all models, suggests there is little rate variation between known branches of the MTBC. Nevertheless, the relaxed clock appeared to have a slightly better performance (Table 2). To experiment with models that allowed for dynamic populations, we applied a Bayesian skyline coalescent (SKY+UCLD) and birth-death skyline prior (BDSKY+UCLD) combined with a relaxed clock model. In the BDSKY+UCLD model, the tree was conditioned on the root. In a prior study, Kühnert and colleagues used birth-death tree priors to investigate two modern tuberculosis outbreaks [38]. To our knowledge, this study is the first to use a birth-death tree prior to infer evolutionary dynamics of the MTBC while using ancient data for tip calibration. The BDSKY+UCLD model had the highest marginal likelihood value of all models applied to this dataset (Table 2).

**Table 2:** Model comparison for full MTBC dataset

Model	Marginal likelihood	Mean rate (95% HPD)	Mean rate variance (95% HPD)	Mean tree height (95% HPD)
BDSKY+UCLD	− 6125044.47176458	1.4488E−8 (9.4606E−9, 1.9632E−8)	1.6881E−17 (5.4855E−18, 3.069E−17)	3258.0478 (2189.5235, 4501.1384)
CC+UCLD	− 6126017.15694528	1.214E−8 (7.1934E−9, 1.6448E−8)	1.2459E−17 (2.833E−18, 2.3969E−17)	4172.1961 (2585.2349, 6119.744)
SKY+UCLD	− 6127733.35000634	1.2944E−8 (8.6149E−9, 1.7342E−8)	1.3423E−17 (4.848E−18, 2.3869E−17)	3650.4222 (2472.6434, 4992.0277)
CC+strict	− 6125541.68118691	1.1573E−8 (8.6397E−9, 1.4509E−8)	NA	4453.1162 (3330.1516, 5619.3974)

Marginal likelihood and parameter estimates from four models applied to the full MTBC dataset: constant coalescent with uncorrelated lognormal clock (CC+UCLD), constant coalescent with strict clock (CC+strict), Bayesian skyline coalescent with uncorrelated lognormal clock (SKY+UCLD), and birth-death skyline with uncorrelated lognormal clock (BDSKY+UCLD). Marginal likelihoods obtained through path sampling (see the “Methods” section)

**Fig. 3:**
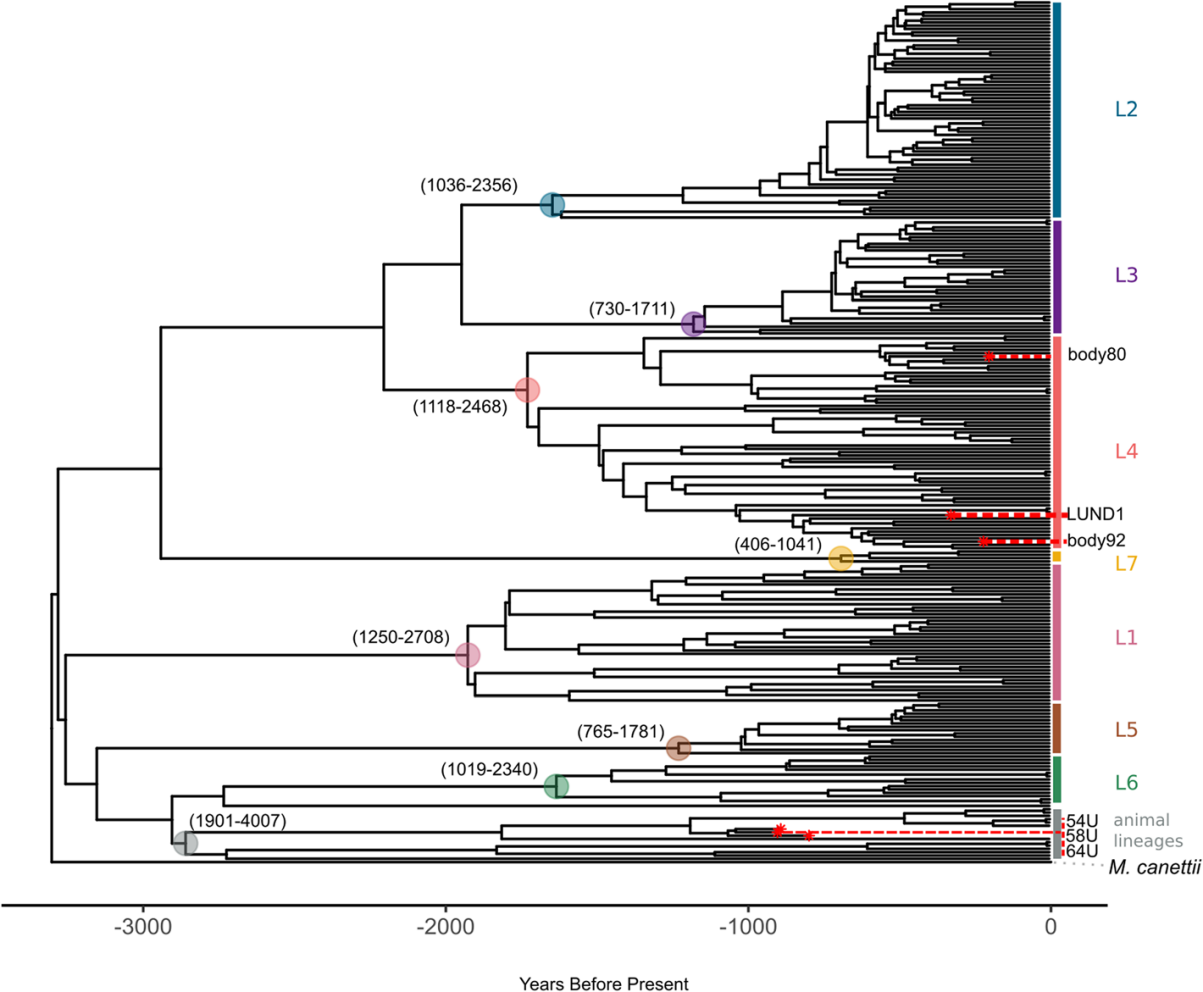
MTBC maximum clade credibility tree. This MCC tree of mean heights was generated from the BDSKY+UCLD model as applied to the full MTBC dataset. Lineages are labeled on the right side. The ancient genomes are indicated by red asterisks and labeled on the side with their sample names. The outgroup is labeled as “*M*. *canettii*.” The 95% HPD intervals of the heights of nodes ancestral to each lineage are indicated as (lower boundary–upper boundary) in years before present. Ancestral nodes are highlighted by a circle colored to match the lineage label. The time scale is expressed as years before present, with the most recent time as 2010. The accompanying skyline plot can be found in Fig. S10 in Additional File 3

A calibrated maximum clade credibility (MCC) tree was generated for the BDSKY+UCLD model, with 3258 years before present (BP) (95% highest posterior density [95% HPD] interval, 2190–4501 BP) as an estimated date of emergence for the MTBC (Fig. 3a). Tree topology agrees with previously presented phylogenetic analyses of the full MTBC [3, 5, 39]. To test the meaningfulness of our ancient tip calibrations, we performed a date randomization test of this model in which we randomly shuffled the tip dates among the genomes in the dataset ten times and compared the clock rate estimates with the randomized models to that of the “true” BDSKY+UCLD model for the MTBC dataset [40, 41]. For this dataset, the tip shuffling caused extremely slow convergence. Though only four out of ten randomized models reached an ESS of over 200 for the clock rate parameter, all randomizations reached an ESS of 100 or greater with combined chain lengths of over 1,000,000,000 (Additional File 10). Date randomizations are evaluated based on two criteria of differing stringency: (i) the mean rate estimate of the randomization does not fall within the 95% HPD interval of the original model, or (ii) the 95% HPD interval of the randomization does not overlap with that of the original model [40]. All randomizations for the MTBC dataset fulfilled the more stringent criteria ii, indicating the tip calibrations from the ancient genomes firmly informed our results (Additional File 10; Fig. S9 in Additional File 3).

The L4 dataset includes LUND1 and the two Hungarian mummies described above [6] as calibration points. We selected 149 modern genomes representative of the known diversity of L4 from previously published datasets (Additional File 3) [3, 21, 31]. A modern Lineage 2 (L2) genome was used as an outgroup. After the exclusion of sites as discussed above (Additional File 8), a SNP alignment of these genomes in reference to the reconstructed TB ancestor genome [21] included a total of 17,333 variant positions, excluding positions unique to the L2 outgroup. Only fifteen variant sites were lost from the L4 dataset alignment. After sites missing from any alignment in the dataset were excluded from downstream analysis, 10,009 SNPs remained for phylogenetic inference. A total of 810 SNPs were identified in LUND1, of which 126 were unique to this genome. A SNP effect analysis [42] was subsequently performed on these derived positions (Additional File 3; Additional File 9). We also assessed the L4 dataset for clock-like structure with TempEst (*R*^2^ = 0.113; see the “Methods” section; Fig. S9 in Additional File 3).

We applied the same models as described above for the full MTBC dataset, with the addition of a birth-death skyline model conditioned on the origin of the root (BDSKY+UCLD+origin). All mean tree heights are within 250 years of each other and the 95% HPD intervals largely overlap. The BDSKY+UCLD and BDSKY+UCLD+origin models show the highest marginal likelihood values after stepping stone sampling. We employed the BDSKY+UCLD+origin model to determine if the estimated origin of the L4 dataset agreed with the tree height estimates for the full MTBC dataset. Intriguingly, the estimated origin parameter (Table 3), or the ancestor of the tree root, largely overlaps with the 95% HPD range for MTBC tree height as seen in Table 2.

**Table 3:** Model comparison for L4 dataset

Model	Marginal likelihood	Mean rate (95% HPD)	Mean rate variance (95% HPD)	Mean tree height (95% HPD)	Origin (BDSKY only)
BDSKY+UCLD	− 6033864.2003	3.1885E−8 (1.9488E−8, 4.4007E−8)	4.991E−17 (1.0674E−17, 8.9835E−17)	1444.5416 (929.3966, 2083.7636)	NA
BDSKY+UCLD+origin	− 60327945.1483	3.4761E−8 (2.447E−8, 4.5029E−8)	5.5123E−17 (1.9718E−17, 9.4555E−17)	1319.2463 (952.8702, 1761.4382)	2310.916 (1165.2155, 3372.9253)
CC+UCLD	− 6043356.1504	3.1068E−8 (1.988E−8, 4.1624E−8)	4.3865E−17 (1.3291E−17, 7.806E−17)	1569.0512 (1054.607, 2225.4758)	NA
SKY+UCLD	− 6034698.3620	2.8097E−8 (1.5329E−8, 3.9927E−8)	3.7609E−17 (6.0593E−18, 7.1919E−17)	1690.536 (1016.2712, 2646.5163)	NA
CC+strict	− 6034091.5119	2.9299E−8 (2.2173E−8, 3.6637E−8)	NA	1567.544 (1186.1186, 1978.6488)	NA

Selected parameter estimates from five models applied to the Lineage 4 dataset: constant coalescent with uncorrelated lognormal clock (CC+UCLD), constant coalescent with strict clock (CC+strict), Bayesian skyline coalescent with uncorrelated lognormal clock (SKY+UCLD), birth-death skyline with uncorrelated lognormal clock and tree conditioned on the root (BDSKY+UCLD), and birth-death skyline with uncorrelated lognormal clock with origin parameter estimate (BDSKY+UCLD+origin). Marginal likelihoods obtained through path sampling (see the “Methods” section)

**Fig. 4:**
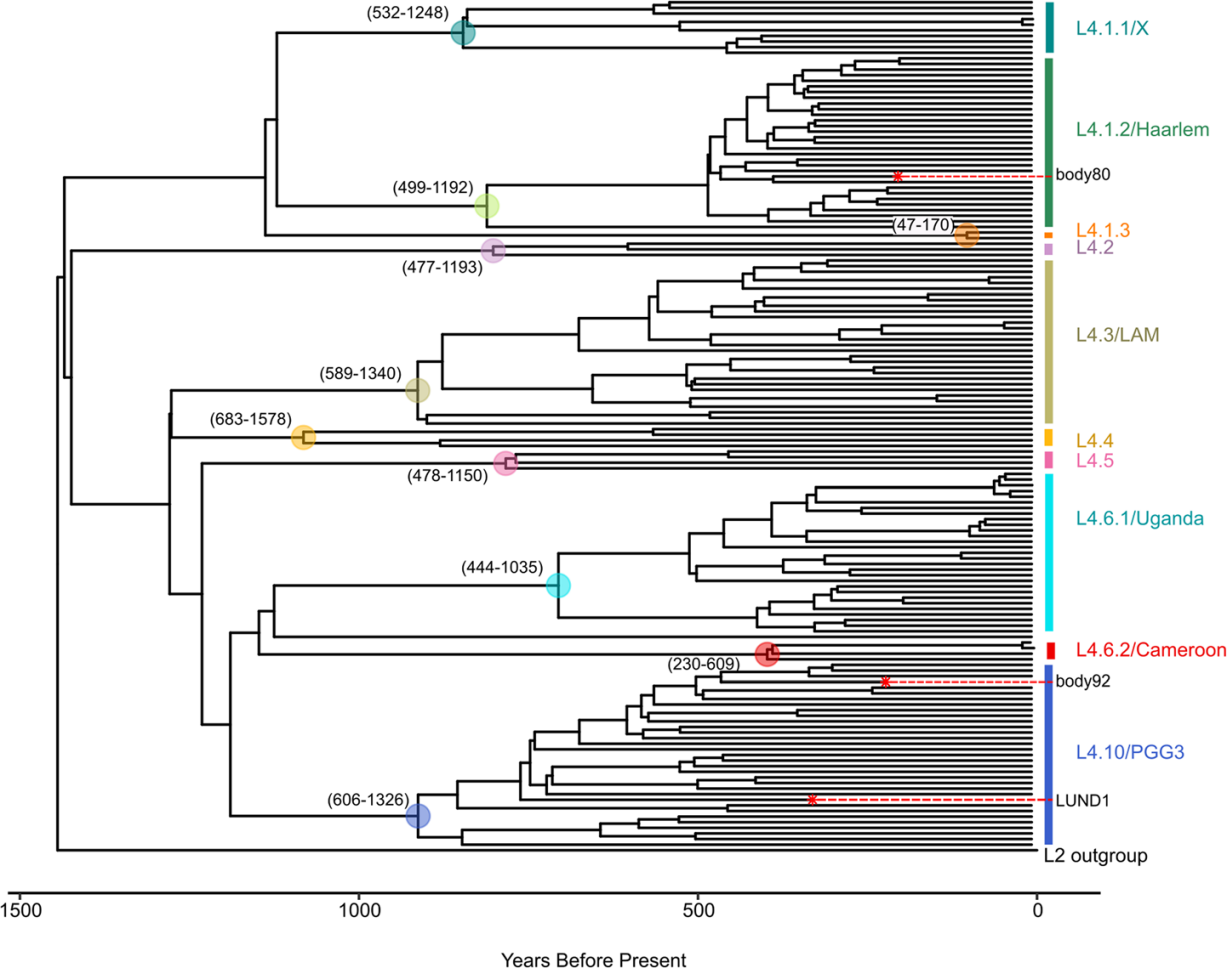
L4 maximum clade credibility tree. This MCC tree of mean heights was generated from the BDSKY+UCLD model as applied to the L4 dataset. Sublineages are labeled on the right side. The ancient genomes are indicated by red asterisks and labeled with their sample name. The Lineage 2 outgroup, represented by L2_N0020, is labeled on the side. The 95% HPD interval for node height is displayed for ancestral nodes of each sublineage as (lower boundary–upper boundary) in years before present. Ancestral nodes are highlighted by a circle colored to match the sublineage label. The time scale is expressed as years before present, with the most recent time as 2010. The accompanying skyline plot can be found in Fig. S13 in Additional File 3

A calibrated MCC tree (Fig. 4) was generated based on the BDSKY+UCLD model for the L4 dataset. This model yielded an estimated date of emergence for L4 of 1445 BP (95% HPD, 929–2084 BP). The tree reflects the ten-sublineage topology presented by Stucki and colleagues [31], with LUND1 grouping with the L4.10/PGG3 sublineage. Due to the relatively low *R*^2^ value for the relationship between sampling time and root-to-tip distance as calculated using TempEst, we also performed a date randomization test of the L4 BDSKY+UCLD model, in which we shuffled the sampling dates randomly among all genomes [40, 41]. We performed ten randomizations and compared the resulting clock rate estimates with that of the BDSKY+UCLD model with the true sampling dates (Table 3). Nine out of ten randomizations fulfilled the more stringent criterion ii, exhibiting no overlap between their 95% HPD intervals and that of the original (Additional File 11; Fig. S12 in Additional File 3). All ten randomizations satisfied criterion i (i.e., yielded a mean rate estimate that fell outside the 95% HPD interval of the rate from the model using true temporal values).

## Discussion

The increasing number of ancient *Mycobacterium tuberculosis* genomes is steadily reducing the uncertainty of molecular dating estimates for the emergence of the MTBC. Here, using the ancient data available to date, we directly calibrate the MTBC time tree and confirm that known diversity within the complex is derived from a common ancestor that existed ~ 2000–6000 years before present (Fig. 3; Table 2) [5, 6]. Our results support the hypothesis that the MTBC emerged during the Neolithic, and not before. The Neolithic revolution generally refers to the worldwide transition in lifestyle and subsistence from more mobile, foraging economies to more sedentary, agricultural economies made possible by the domestication of plants and animals. The period during which it occurred varies between regions. In Africa, where the MTBC is thought to have originated [3, 43–45], the onset of these cultural changes, and animal domestication in particular, appears to have its focus around ~ 3000 BCE, or 5000 BP, across multiple regions [46]. The estimates presented here place the emergence of tuberculosis amidst the suite of human health impacts that took place as a consequence of the Neolithic lifestyle changes often referred to collectively as the first epidemiological transition [47, 48].

Tuberculosis has left testaments to its history as a human pathogen in the archeological record [49], where some skeletal analyses have been interpreted to suggest tuberculosis in human and animal remains pre-dating the upper 95% HPD boundary for the MTBC tMRCA presented here [7, 8, 10, 50–54]. However, it is important to explore the evolutionary history of the MTBC through molecular data. Furthermore, it is crucial to base molecular dating estimates on datasets that include ancient genomes, which expand the temporal sampling window and provide data from the pre-antibiotic era. Numerous studies have found long-term nucleotide substitution rate estimates in eukaryotes and viruses to be dependent on the temporal breadth of the sampling window, and it is reasonable to assume the same principle applies to bacteria [55–60]. Additionally, rate variation over time and between lineages, which may arise due to changing evolutionary dynamics such as climate and host biology, can impact the constancy of the molecular clock [58, 59]. Though models have been developed to accommodate uncertainty regarding these dynamics [61], temporally structured populations can provide evidence and context for these phenomena over time and can aid researchers in refining models appropriate for the taxon in question [60]. Though we did not identify substantial rate variation within either the MTBC or L4 trees (Figs. S14 and S15 in Additional File 3), it is important that we draw these observations from temporally structured datasets and continue to do so in the future.

In addition to our tMRCA estimate for the MTBC, we present one for L4, which is among the most globally dominant lineages in the complex [31, 62]. Our analyses yielded tMRCA dates between ~ 900 and 2500 years before present, as extrapolated from the 95% HPD intervals of all models (Table 3), with mean dates spanning from 320 to 691 CE. These results are strikingly similar to those found in two prior publications and support the idea proposed by Kay and colleagues that L4 may have emerged during the late Roman period [5, 6]. However, there exist discrepancies between different estimates for the age of this lineage in available literature that overlap with the upper [63] and lower [62] edges of the 95% HPD intervals reported here. In addition, recent phylogeographic analyses of the MTBC and its lineages had ambiguous results for L4, with the internal nodes being assigned to either African or European origins depending on the study or different dataset structures used within the same study [62, 63]. This finding indicates a close relationship between ancestral L4 strains in Europe and Africa [62, 63]. Stucki and colleagues delineated L4 into two groups based on the extent of their geographic distribution: globally distributed “generalist” sublineages and highly local “specialist” sublineages that do not appear outside a restricted geographical niche [31]. Thus far, the “specialist” sublineages are found regionally on the African continent. A clear phylogenetic relationship explaining the distinction between geographically expansive and limited strains has not been established. Specifically, LUND1 falls within the globally distributed, “generalist” L4.10/PGG3 sublineage that shares a clade with two “specialist” sublineages: L4.6.1/Uganda and L4.6.2/Cameroon (Fig. 4) [31]. Elucidating the phenomenon that separated L4.10/PGG3 and the L4.6 lineages could offer relevant clues about the evolutionary relationship between specific populations of MTBC organisms and specific populations of humans by selection or genetic drift discussed elsewhere [44, 64]. Assuming modern L4 diversity in Africa was driven by exchanges between Europe and Africa [62, 63], why do we not see the L4.6 lineages more frequently in European populations as we do their sister clade? The current discrepancies over the age and geographic origin of L4 make interpretations of existing data unreliable for questions of such specificity and complexity at this time. These discrepancies could be due to differences in genome selection, SNP selection, and/or model selection and parameterization. It is unlikely we will gain clarity until more diverse, high-quality ancient L4 genomes are generated, creating a more temporally and geographically structured dataset.

**Fig. 5:**
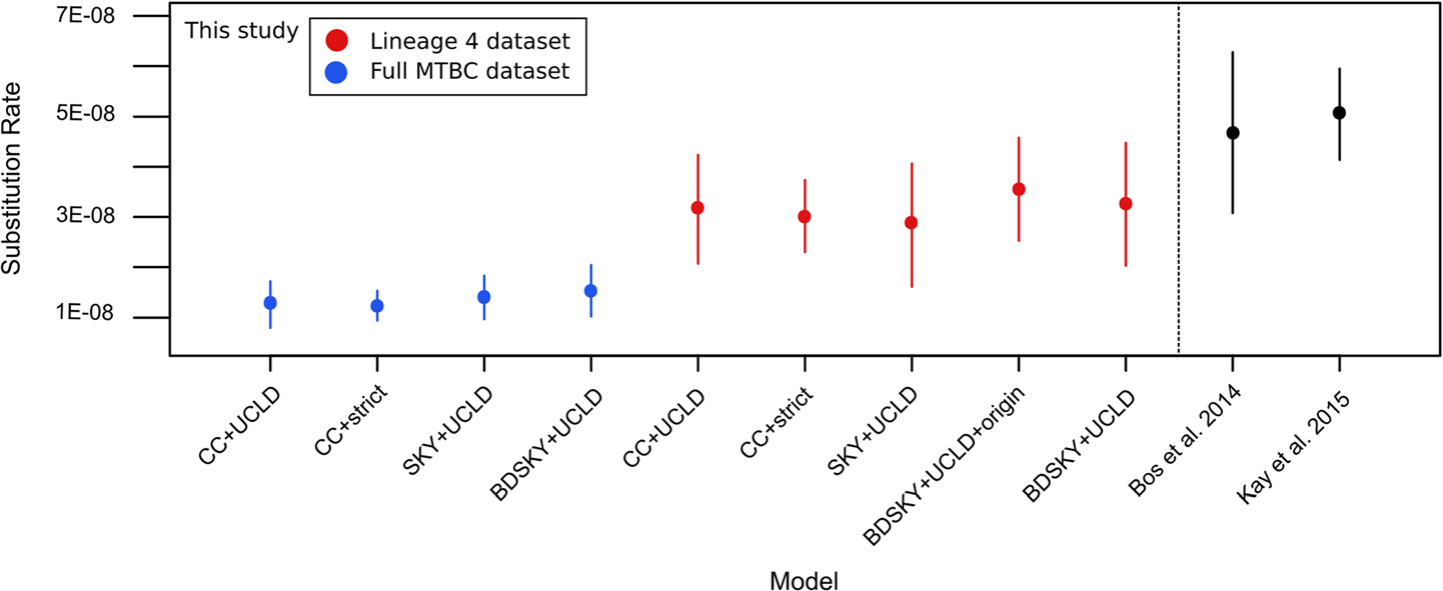
Substitution rate comparison across models and studies. Mean substitution rate per site per year for all models is expressed by a filled circle, with extended lines indicating the 95% HPD interval for that parameter. The Bos et al. [5] and Kay et al. [6] ranges are based on the reported rate values in each study. The Bos et al. [5] range is based on a full MTBC dataset, while the Kay et al. [6] range is based on an L4 dataset. All values presented here fall within one order of magnitude

Going deeper into comparisons between the results presented here and those from prior studies, mutation rate estimates in the L4 and full MTBC analyses were lower than previous estimates for comparable datasets, but within the same order of magnitude, with all mean and median estimates ranging between 1E−8 and 5E−8 [5, 6] (Table 2). Nucleotide substitution rates inferred based on modern tuberculosis data are close to but slightly higher than those based on ancient calibration, with multiple studies finding rates of approximately 1E−7 substitutions per site per year [4, 65]. Despite a strict clock model having been rejected by the MEGA-CC molecular clock test [66] for both the L4 and full MTBC datasets, the clock rate variation estimates do not surpass 9E−17 in any model. Additionally, there is little difference between the clock rates estimated in the L4 and full MTBC datasets suggesting the rate of evolution in L4 does not meaningfully differ from that of the full complex (Tables 2 and 3; Fig. 5).

Importantly, we explored our data through multiple models, including birth-death tree priors. In our opinion, these models offer more robust parameterization options for heterochronous datasets that are unevenly distributed over time, such as those presented here, by allowing for uneven sampling proportions across different time intervals of the tree [67]. Recent studies have demonstrated the importance of selecting appropriate tree priors for the population under investigation, as well as the differences between birth-death and coalescent tree priors [68, 69]. It is notable that the estimates reported here roughly agree across multiple demographic and clock models implemented in BEAST2. The estimate of the origin height for the L4 dataset as calculated with the birth-death Skyline model overlaps with the 95% HPD intervals for the tree height estimates across models in the full MTBC dataset.

In addition to confirming the findings of prior publications, this study contributes a high-coverage, contamination-free, and securely dated ancient *M*. *tuberculosis* genome for future dating efforts, which may include more ancient data or more realistic models. Much of this quality likely comes from the unique preservation environment of the calcified nodule. In the case of tuberculosis, such nodules form from host immunological responses in the waning period of an active pulmonary infection [70] and remain in lung tissue, characterizing the latent form of the disease. Host immune cells were likely responsible for the dominant signal of human DNA in the LUND1 metagenomic screening library (Fig. 1, Supplementary Table 2 in Additional File 1). Similar levels of preservation have been observed through analyses of ancient nodules yielding *Brucella* [71] and urogenital bacterial infections [72], with pathogen preservation surpassing what we report here.

LUND1 avoided multiple quality-related problems often encountered in the identification and reconstruction of ancient genetic data from the MTBC. The genome is of high quality both in terms of its high coverage and low heterozygosity. Despite the low quantity of MTBC DNA detected in the preliminary screening data, in-solution capture enriched the proportion of endogenous DNA by three orders of magnitude (Table 1). The resultant genomic coverage left few ambiguous positions at which multiple alleles were represented by greater than 10% of the aligned reads. This extremely low level of heterozygosity indicated that LUND1 contained a dominant signal of only one MTBC strain. This circumvented analytical complications that can arise from the simultaneous presence of multiple MTBC strains associated with mixed infections or from the presence of abundant non-MTBC mycobacteria stemming from the environment. The preservation conditions of Bishop Winstrup’s remains, mummified in a crypt far from soil, left the small MTBC signal unobscured by environmental mycobacteria or by the dominance of any other bacterial organisms (Fig. 2a). The unprecedented quality of LUND1 and the precision of its calibration point (historically recorded year of death) made it ideal for Bayesian molecular dating applications.

While the high quality and securely dated ancient genome presented here offered advantages in a molecular dating approach, there are caveats to the results of this study. First, this analysis excludes diversity within *M*. *canettii*—a bacterium that can cause pulmonary tuberculosis—from the MTBC dataset, and as such, our estimate does not preclude the possibility of a closely related ancestor having caused infections indistinguishable from tuberculosis in humans before 6000 BP. The inferred tMRCA could be restricted to a lineage that survived an evolutionary bottleneck or selective sweep, possibly connected to its virulence in humans as suggested elsewhere, albeit as a considerably more ancient event [45, 73, 74]. It is possible there were pathogenic sister lineages to the MTBC that existed prior to this reduction in diversity and are not represented by extant MTBC diversity. Additionally, despite the use of ancient data, our temporal sampling window is still narrow given the estimated age of the MTBC and L4. For the MTBC dataset no samples pre-date 1000 years before present, and for L4, no samples predate 350 years before present. It could be argued the ancient L4 genomes available to date represent samples taken in the midst of an epidemic—namely, the “White Plague” of tuberculosis, which afflicted Europe between the seventeenth and nineteenth centuries [75]. For a slow-evolving bacterial pathogen like tuberculosis, it is possible our sampling window of ancient genomes is subject to the very issue they are meant to alleviate: the time dependency of molecular clocks [55, 57–59]. The genomes sampled from pre-contact Peruvian remains do not derive from a known epidemic period in history and add temporal spread to our MTBC dataset, but also belong to a clade of animal-associated strains (*M*. *pinnipedii*) that may have been subject to dramatically different evolutionary pressures compared to the human-associated lineages of the complex due to differing host biology and population dynamics. However, our use of a relaxed clock model allowed for the estimation and accommodation of variable rates across different branches of the complex. We do not see evidence for divergent substitution rates among the branches leading to the Peruvian *M*. *pinnipedii* strains (Fig. S14 in Additional File 3). On a related matter, we may be missing diversity for some lineages (e.g., L6, L7, animal lineages) for which whole genome data is sparse. The available ancient MTBC genomes also suffer from a lack of lineage diversity, with only pinniped strains and L4 represented. We furthermore qualify our BDSKY results by acknowledging our models required the specification of priors for the *rho* parameter (the sampling proportion of the total population at discrete time points). We chose *rho* priors (see Additional File 3) assuming that our modern genomes represented a greater sampling proportion of the total contemporaneous MTBC and L4 populations than our ancient genomes. This assumption alone made this parameterization less arbitrary than the assumptions inherent in the coalescent-based methods that have been utilized in the past for similar time-sampled analyses of the MTBC and other pathogens, which assume random sampling at uniform rates across all time periods. We also acknowledge that skyline models assume panmictic populations, and the datasets presented here do contain spatial subdivision, which may bias estimates regarding population dynamics. However, this aspect of our datasets is unlikely to bias our molecular clock estimates. As stated above, the agreement of multiple models to reach similar dates for the tMRCA of the MTBC and L4 reinforces our support of the hypothesis that the most recent common ancestor of the MTBC diversity we are aware of today emerged during the Neolithic.

Filling the MTBC time tree with more ancient genomes from diverse time periods, locations, and lineages would have the potential to address the limitations listed above. The most informative data would (a) derive from an Old World context (i.e., Europe, Asia, or Africa) pre-dating the White Plague in Europe or (b) come from any geographical location or pre-modern time period, but belong to one of the MTBC lineages not yet represented by ancient data. An ideal data point, which would clarify many open questions and seeming contradictions related to the evolutionary history of the MTBC, would derive from Africa, the inferred home of the MTBC ancestor [3, 43–45], and pre-date 2000 years before present. A genome of this age would test the lower boundaries of the 95% HPD tree height intervals estimated in the full MTBC models presented here. Until recently, it would have been considered unrealistic to expect such data to be generated from that time period and location. Innovations and improvements in ancient DNA retrieval and enrichment methods, however, have brought this expectation firmly into the realm of the possible [30, 76]. Ancient bacterial pathogen genomes have now been retrieved from remains from up to 5000 years before present [77–79] and recent studies have reported the recovery of human genomes from up to 15,000-year-old remains from North Africa [80, 81].

## Conclusions

Here, we offer confirmation that the extant MTBC, and all available ancient MTBC genomes, stem from a common ancestor that existed a maximum of 6000 years before present. Many open questions remain, however, regarding the evolutionary history of the MTBC and its constituent lineages, as well as the role of tuberculosis in human history. Elucidating these questions is an iterative process, and progress will include the generation of diverse ancient *M*. *tuberculosis* genomes, and the refinement and improved parameterization of Bayesian models that reflect the realities of MTBC (and other organisms’) population dynamics and sampling frequencies over time. To aid in future attempts to answer these questions, this study provides an ancient MTBC genome of impeccable quality and explores the first steps in applying birth-death population models to modern and ancient TB data.

## Methods

### Lung nodule identification

The paleopathological investigation of the body of Winstrup is based on extensive CT scan examinations with imaging of the mummy and its bedding performed with a Siemens Somatom Definition Flash, 128 slice at the Imaging Department of Lund University Hospital. Ocular inspection of the body other than of the head and hands was not feasible, since Winstrup was buried in his episcopal robes and underneath the body was wrapped in linen strips. The velvet cap and the leather gloves were removed during the investigation. The body was naturally mummified and appeared to be well preserved with several internal organs identified.

The imaging was quite revealing. The intracranial content was lost with remains of the brain in the posterior skull base. Further, the dental status was poor with several teeth in the upper jaw affected by severe attrition, caries, and signs of tooth decay, as well as the absence of all teeth in the lower jaw. Most of the shed teeth were represented by closed alveoli, indicating antemortem tooth loss. Along with the investigation of the bedding, a small sack made of fabric was found behind the right elbow containing five teeth: two incisors, two premolars, and one molar. The teeth in the bag complemented the remaining teeth in the upper jaw. It is feasible that the teeth belonged to Winstrup and were shed several years before he died. A fetus approximately 5 months of age was also found in the bedding, underneath his feet.

Both lungs were preserved but collapsed with findings of a small parenchymal calcification and two ~ 5 mm calcifications in the right hilum (Fig. 1). The assessment was that these could constitute a Ranke complex, suggestive of previous primary tuberculosis [70]. A laparoscopy was performed at the Lund University Hospital in a clinical environment whereby the nodules were retrieved. Furthermore, several calcifications were also found in the aorta and the coronary arteries, suggesting the presence of atherosclerosis. The stomach, liver, and gall bladder were preserved, and several small gallstones were observed. The spleen could be identified but not the kidneys. The intestines were there, however, collapsed except for the rectum that contained several large pieces of concernments. The bladder and the prostate could not be recognized.

The skeleton showed several pathological changes. Findings on the vertebrae consistent with DISH (diffuse idiopathic skeletal hyperostosis) were present in the thoracic and the lumbar spine. Reduction of the joint space in both hip joints and the left knee joint indicate that Winstrup was affected by osteoarthritis. No signs of gout or osteological tuberculosis (i.e., Pott’s disease) were found.

Neither written sources nor the modern examination of the body of Winstrup reveal the immediate cause of death. However, it is known that he was bedridden for at least 2 years preceding his death. Historical records indicate that gallstones caused him problems while traveling to his different parishes. Additionally, he was known to have suffered from tuberculosis as a child, which may have recurred in his old age.

### Sampling and extraction

Sampling of the lung nodule, extraction, and library preparation were conducted in dedicated ancient DNA clean rooms at the Max Planck Institute for the Science of Human History in Jena, Germany. The nodule was broken using a hammer, and a 5.5 mg portion of the nodule was taken with lung tissue for extraction according to a previously described protocol with modifications [18]. The sample was first decalcified overnight at room temperature in 1 mL of 0.5 M EDTA. The sample was then spun down, and the EDTA supernatant was removed and frozen. The partially decalcified nodule was then immersed in 1 mL of a digestion buffer with final concentrations of 0.45 M EDTA and 0.25 mg/mL Proteinase K (Qiagen) and rotated at 37 °C overnight. After incubation, the sample was centrifuged. The supernatants from the digestion and initial decalcification step were purified using a 5-M guanidine-hydrochloride binding buffer with a High Pure Viral Nucleic Acid Large Volume kit (Roche). The extract was eluted in 100 μl of a 10-mM tris-hydrochloride, 1-mM EDTA (pH 8.0), and 0.05% Tween-20 buffer (TET). Two negative controls and one positive control sample of cave bear bone powder were processed alongside LUND1 to control for reagent/laboratory contamination and process efficiency, respectively.

### Library preparation and shotgun screening sequencing

Double-stranded Illumina libraries were constructed according to an established protocol with some modifications [82]. Overhangs of DNA fragments were blunt-end repaired in a 50 μl reaction including 10 μl of the LUND1 extract, 21.6 μl of H_2_O, 5 μl of NEB Buffer 2 (New England Biolabs), 2 μl dNTP mix (2.5 mM), 4 μl BSA (10 mg/ml), 5 μl ATP (10 mM), 2 μl T4 polynucleotide kinase, and 0.4 μl T4 polymerase, then purified and eluted in 18 μl TET. Illumina adapters were ligated to the blunt-end fragments in a reaction with 20 μl Quick Ligase Buffer, 1 μl of adapter mix (0.25 μM), and 1 μl of Quick Ligase. Purification of the blunt-end repair and adapter ligation steps was performed using MinElute columns (Qiagen). Adapter fill-in was performed in a 40-μl reaction including 20 μl adapter ligation eluate, 12 μl H_2_O, 4 μl Thermopol buffer, 2 μl dNTP mix (2.5 mM), and 2 μl Bst polymerase. After the reaction was incubated at 37 °C for 20 min, the enzyme was heat deactivated with a 20-min incubation at 80 °C. Four library blanks were processed alongside LUND1 to control for reagent/laboratory contamination. The library was quantified using a real-time qPCR assay (Lightcycler 480 Roche) with the universal Illumina adapter sequences IS7 and IS8 as targets. Following this step, the library was double indexed [83] with a unique pair of indices over two 100 μl reactions using 19 μl of template, 63.5 μl of H_2_O, 10 μl PfuTurbo buffer, 1 μl PfuTurbo (Agilent), 1 μl dNTP mix (25 mM), 1.5 μl BSA (10 mg/ml), and 2 μl of each indexing primer (10 μM). The master mix was prepared in a pre-PCR clean room and transported to a separate lab for amplification. The two reactions were purified and eluted in 25 μl of TET each over MinElute columns (Qiagen), then assessed for efficiency using a real-time qPCR assay targeting the IS5 and IS6 sequences in the indexing primers. The reactions were then pooled into one double-indexed library. Approximately one third of the library was amplified over three 70 μl PCR reactions using 5 μl of template each and Herculase II Fusion DNA Polymerase (Agilent). The products were MinElute purified, pooled, and quantified using an Agilent Tape Station D1000 Screen Tape kit. LUND1 and the corresponding negative controls were sequenced separately on an Illumina NextSeq 500 using single-end, 75-cycle, high-output kits.

### Pathogen identification and authentication

De-multiplexed sequencing reads belonging to LUND1 were processed in silico with the EAGER pipeline (v.1.92) [24]. ClipAndMerge was used for adapter removal, fragment length filtering (minimum sequence length, 30 bp), and base sequence quality filtering (minimum base quality, 20). MALT v. 038 [19] was used to screen the metagenomic data for pathogens using the full NCBI Nucleotide database (“nt,” April 2016) with a minimum percent identity of 85%, a minSupport threshold of 0.01, and a topPercent value of 1.0. The resulting metagenomic profile was visually assessed with MEGAN6 CE [20]. The adapter-clipped reads were additionally aligned to a reconstructed MTBC ancestor genome [21] with BWA [33] as implemented in EAGER (-l 1000, -n 0.01, -q 30). Damage was characterized with DamageProfiler in EAGER [84].

### In-solution capture probe design

Single-stranded probes for in-solution capture were designed using a computationally extrapolated ancestral genome of the MTBC [21]. The probes are 52 nucleotides in length with a tiling density of 5 nucleotides, yielding a set of 852,164 unique probes after the removal of duplicate and low complexity probes. The number of probes was raised to 980,000 by a random sampling among the generated probe sequences. A linker sequence (5′-CACTGCGG-3′) was attached to each probe sequence, resulting in probes of 60 nucleotides in length, which were printed on a custom-design 1 million-feature array (Agilent). The printed probes were cleaved off the array, biotinylated, and prepared for capture according to Fu et al. [30].

### UDG library preparation and in-solution capture

Fifty microliters of the original LUND1 extract were used to create a uracil-DNA glycosylase (UDG) treated library, in which the post-mortem cytosine to uracil modifications, which cause characteristic damage patterns in ancient DNA, are removed. The template DNA was treated in a buffer including 7 μl H_2_O, 10 μl NEB Buffer 2 (New England Biolabs), 12 μl dNTP mix (2.5 mM), 1 μl BSA (10 mg/ml), 10 μl ATP (10 mM), 4 μl T4 polynucleotide kinase, and 6 μl USER enzyme (New England Biolabs). The reaction was incubated at 37 °C for 3 h, and then 4 μl of T4 polymerase was added to the library to complete the blunt-end repair step. The remainder of the library preparation protocol, including double indexing, was performed as described above.

The LUND1 UDG-treated library was amplified over two rounds of amplification using Herculase II Fusion DNA Polymerase (Agilent). In the first round, five reactions using 3 μl of template each were MinElute purified and pooled together. The second round of amplification consisted of three reactions using 3 μl of template each from the first amplification pool. The resulting products were MinElute purified and pooled together. The final concentration of 279 ng/μl was measured using an Agilent Tape Station D1000 Screen Tape kit (Agilent). A portion of the non-UDG library (see above) was re-amplified to 215 ng/μl. A 1:10 pool of the non-UDG and UDG amplification products was made to undergo capture. A pool of all associated negative control libraries (Supplementary Table 2) and a positive control known to contain *M*. *tuberculosis* DNA also underwent capture in parallel with the LUND1 libraries. Capture was performed according to an established protocol [29], and the sample product was sequenced on an Illumina NextSeq with a 150-cycle paired end kit to a depth of ~ 60 million paired reads. The negative controls were sequenced on a NextSeq 500 with a 75-cycle paired end kit.

### Genomic reconstruction, heterozygosity, and SNP calling

For the enriched, UDG-treated LUND1 sequencing data, de-multiplexed paired-end reads were processed with the EAGER pipeline (v. 1.92) [24], adapter-clipped with AdapterRemoval, and aligned to the MTBC reconstructed ancestor genome with BWA (-l 32, -n 0.1, -q 37). Previously published ancient and modern *Mycobacterium tuberculosis* genomic data (Supplementary Table 4, Supplementary Table 5) were processed as single-end sequencing reads, but otherwise processed identically in the EAGER pipeline. Genome Analysis Toolkit (GATK) UnifiedGenotyper was used to call SNPs using default parameters and the EMIT ALL SITES output option [85]. We used MultiVCFAnalyzer (v0.87 https://github.com/alexherbig/MultiVCFAnalyzer) [5] to create and curate SNP alignments for the L4 (Supplementary Table 5) and full MTBC (Supplementary Table 4) datasets based on SNPs called in reference to the TB ancestor genome [21], with repetitive sequences, regions subject to cross-species mapping, and potentially imported sites excluded. The repetitive and possibly cross-mapped regions were excluded as described previously [5]. Potentially imported sites were identified using ClonalFrameML [34] separately for each dataset, using full genomic alignments and trees generated in RAxML [86] as input without the respective outgroups. Remaining variants were called as homozygous if they were covered by at least 5 reads, had a minimum genotyping quality of 30, and constituted at least 90% of the alleles present at the site. Outgroups for each dataset were included in the SNP alignments, but no variants unique to the selected outgroup genomes were included. Minority alleles constituting over 10% were called and assessed for LUND1 to check for a multiple strain *M*. *tuberculosis* infection. Sites with missing or incomplete data were excluded from further analysis.

### Phylogenetic analysis

Neighbor joining (Figs. S2 and S3 in Additional File 3), maximum likelihood (Figs. S4 and S5 in Additional File 3), and maximum parsimony (Figs. S6 and S7 in Additional File 3) trees were generated for the L4 and full MTBC datasets (Tables S4 and S5 in Additional File 1), with 500 bootstrap replications per tree. Maximum parsimony and neighbor joining trees were configured using MEGA-Proto and executed using MEGA-CC [66]. Maximum likelihood trees were configured and executed using RAxML [86] with the GTR+GAMMA (4 gamma categories) substitution model.

### Bayesian phylogenetic analysis of full MTBC and L4 datasets

Bayesian phylogenetic analysis of the full MTBC was conducted using a dataset of 261 *M*. *tuberculosis* genomes including LUND1, five previously published ancient genomes [5, 6], and 255 previously published modern genomes (Additional File 5). *Mycobacterium canettii* was used as an outgroup for this dataset. Bayesian phylogenetic analysis of L4 of the MTBC was conducted using a dataset of 152 genomes including three ancient genomes presented here and in a previous publication [6] and 149 previously published modern genomes (Additional File 6). Body80 and body92 were selected out of the eight samples presented by Kay and colleagues based on multiple criteria. Multiple samples from that study proved to be mixed strain infections. Apart from body92, these samples were excluded from this analysis due to our present inability to separate strains with retention of unique positions. Body92 had a clearly dominant strain estimated by Kay et al. [6] to constitute 96% of the tuberculosis DNA in the sample, and stringent mapping in BWA [33] (-l 32, -n 0.1, -q 37) for this project found the genome to have 124-fold coverage when mapped against the TB ancestor. Between the degree of dominance and the high coverage, we could confidently call variant positions from the dominant strain (Fig. S16a in Additional File 3). Body80 was the only single-strain sample from that collection to have sufficient coverage (~8x) for confident SNP calling after stringent mapping (Fig. S16b in Additional File 3). For selection criteria for the modern genomes, please see Additional File 3. L2_N0020 was used as an outgroup. The possibility of equal evolutionary rates in both datasets was rejected by the MEGA-CC molecular clock test [66]. TempEst [87] was also used to assess temporal structure in the phylogeny prior to analysis with BEAST2 [37]. For the full MTBC alignment, *R*^2^ = 0.273, and for the L4 alignment, *R*^2^ = 0.113 (Figs. S8 and S9 in Additional File 3). We generated a maximum likelihood tree and alignment for the full MTBC excluding the animal-associated lineages, and consequently excluding the ancient *M*. *pinnipedii* genomes, to test if limiting the dataset to the human lineages produced a stronger temporal signal. Without the anchor of the ancient *M*. *pinnipedii* genomes, the temporal signal for the full complex reduced (*R*^2^ = 0.06), as all ancient calibration points were limited to Lineage 4 (Fig. S17 in Additional File 3). When the root-to-tip distances are plotted with points labeled according to lineage or sublineage, it becomes clear that clade membership is largely driving the distance from root of the genomes. However, there remains a temporal signal in the data.

A correction for static positions in the *M*. *tuberculosis* genome not included in the SNP alignment was included in the configuration file. A “TVM” substitution model, selected based on results from ModelGenerator [88], was implemented in BEAUti as a GTR+G4 model with the AG rate parameter fixed to 1.0. LUND1, body80, and body92 were tip-calibrated using year of death, which was available for all three individuals (Additional File 6). The three ancient Peruvian genomes were calibrated using the mid-point of their OxCal ranges (Additional File 5) [5]. We performed tip sampling for all modern genomes excluding the outgroup over a uniform distribution between 1992 and 2010 in all models. The outgroup was fixed to 2010 in every case. All tree priors were used in conjunction with an uncorrelated relaxed lognormal clock model. The constant coalescent model was also used in conjunction with a strict clock model.

Two independent MCMC chains of 200,000,000 iterations minimum were computed for each model. If the ESS for any parameter was below 200 after the chains were combined, they were resumed with additional iterations. The results were assessed in Tracer v1.7.1 with a 10% burn-in [89]. Trees were sampled every 20,000 iterations. The log files and trees for each pair of runs were combined using LogCombiner v2.4.7 [37]. An MCC tree was generated using TreeAnnotator with 10% burn-in [37]. Figures 3 and 4 were generated using the ggtree package [90] in R [91]. For details on the parameterization of the birth-death models, please see Additional File 3.

Marginal likelihood was calculated using stepping stone sampling [92] implemented in the MODELSELECTION package in BEAST2. The total chain length required for convergence in each model was split across 100 steps. Following this, we performed a date randomization test [41] for the BDSKY+UCLD model for each dataset. Dates were shuffled randomly among all genomes excluding the outgroup. For both datasets the outgroup was used as an anchor for tip-dating of the “modern” genomes in each date-randomized model. Ten randomizations were generated for each model and run in at least two parallel chains. For the L4 dataset, the chains were run until the rate parameter reached an ESS of at least 200 for every date-randomized model (Additional File 11). For the date randomizations of the full MTBC dataset, we reached sufficient ESS in four out of ten models. However, as noted above in the “Results” section, we reached ESS values greater than or equal to 100 for the rate parameter for all models. We present the rate estimates and rate parameter ESS values for all MTBC date randomizations (Additional File 10).

## Supplementary information

**Additional file 1: Table S1.** Assigned reads from all taxonomic levels represented in the metagenomic LUND1 library prior to in-solution capture for MTBC DNA.

**Additional file 2: Table S2.** Full EAGER pipeline results for LUND1 shotgun sequencing data when mapped to HG19 human reference genome and TB ancestor genome, the non-UDG-treated enriched LUND1 data when mapped to the TB ancestor genome, and the UDG-treated enriched LUND1 data when mapped to the TB ancestor genome.

**Additional file 3: Supplementary information.** Detailed supplements to the RESULTS and METHODS sections, including supplementary figures.

**Additional file 4: Table S3.** Full EAGER pipeline results for negative controls processed with LUND1, mapped to the reconstructed TB ancestor genome.

**Additional file 5: Table S4.** List of genomes included in the full MTBC dataset, with respective publications, accession numbers, lineages, and dates (when applicable).

**Additional file 6: Table S5.** List of genomes included in the L4 dataset, with respective publications, accession numbers, lineages, dates, and percentage of total SNPs called as heterozygous.

**Additional file 7: Table S6.** List of sites excluded from the full MTBC dataset in GFF format.

**Additional file 8: Table S7.** List of sites excluded from the L4 dataset in GFF format.

**Additional file 9: Table S8.** SnpEff annotation for derived alleles in LUND1.

**Additional file 10: Table S9.** Date randomization results for MTBC dataset.

**Additional file 11: Table S10.** Date randomization results for L4 dataset.

**Additional file 12.** Review history.

## Data Availability

Raw sequencing data generated within this study was uploaded to the NCBI Sequence Read Archive (SRA) (accession SRS6462469; BioProject PRJNA517266) [93]. These data include the non-UDG non-enriched screening library, the non-UDG enriched library, and the UDG-treated enriched library. The previously published data used in the MTBC dataset is available on the SRA and can be accessed as part of the following BioProject accessions: PRJNA244165 [94], PRJEB7454 [95], PRJNA186722 [96], PRJEB3128 [97], PRJEB3223 [98], PRJNA52007 [97], PRJNA39969 [100], PRJEB2138 [101], PRJNA52637 [102], PRJNA38491 [103], PRJNA49659 [104], PRJEB2092 [105], PRJEB2091 [106], and PRJNA244633 [107]. See Additional File 5 for sample-specific accession numbers. The previously published data used in the L4 dataset is available on the SRA and can be accessed as part of the following BioProject accessions: PRJEB7454 [95], PRJEB11460 [108], PRJEB3223 [98], PRJNA52007 [99], PRJNA52637 [102], PRJNA38491 [103], PRJNA39969 [100], and PRJNA49659 [104]. See Additional File 6 for sample-specific accession numbers.
