# Supplementary material for: A seventeenth-century Mycobacterium tuberculosis genome supports a Neolithic emergence of the Mycobacterium tuberculosis complex
Source: Genome Biol. 2020 Aug 10;21:201. doi: 10.1186/s13059-020-02112-1 (PMC7418204; doi:10.1186/s13059-020-02112-1)
Supplement: Supplementary file 3 — Additional file 3: Supplementary information. Detailed supplements to the RESULTS and METHODS sections, including supplementary figures. [file 13059_2020_2112_MOESM3_ESM.docx]

SUPPLEMENTARY INFORMATION

For

A seventeenth-century *Mycobacterium tuberculosis* genome supports a Neolithic emergence of the *Mycobacterium tuberculosis* complex.

**Human DNA and mitochondrial analysis**

De-multiplexed shotgun sequencing reads belonging to LUND1 were processed *in silico* with the EAGER pipeline (v.1.92) (Peltzer et al. 2016). ClipAndMerge was used for adapter removal, fragment length filtering (minimum sequence length: 30 bp), and base sequence quality filtering (minimum base quality: 20). To screen the reads for human DNA preservation, we mapped them to the hg19 human reference genome using BWA (Li and Durbin 2009) as implemented in EAGER with the following parameters: -l 1000, -n 0.01, -q 30. Given the uncharacteristically high endogenous human DNA content of the lung nodule, we estimated the mitochondrial contamination rate using Schmutzi (Renaud et al. 2015). In order to do this we mapped the metagenomic sequencing data to the hg19 mitochondrial chromosome with CircularMapper as implemented in the EAGER pipeline (Peltzer et al. 2016). We called initial contamination estimates with contDeam, and then ran Schmutzi, calling a consensus mitochondrial sequence and making a final average contamination estimate of 2% (lower boundary 1%, upper boundary 3%).

**Heterozygosity analysis and selection of modern L4 genomes**

We chose to reduce the quantity of L4 genomes used for Bayesian phylogeny in this study for the purposes of computational feasibility and attempting to balance the representation of different L4 sublineages without compromising allelic diversity in the analysis. The four deeply sampled sublineages published by Stucki and colleagues (2016) – L4.3/LAM, L4.6.1/Uganda, L4.1.2/Haarlem, and L4.10/PGG3 – were subsampled to 30 representatives each based on the quantity of heterozygous positions found in each genome after the inclusion of strains published in the Comas et al. (2013, 2010) datasets. For each of these four sublineages, the 30 genomes with the fewest heterozygous positions were selected for inclusion. We quantified the number of heterozygous positions using MultiVCFAnalyzer (v0.87 <https://github.com/alexherbig/MultiVCFAnalyzer>) (Bos et al. 2014) (see METHODS).

**SNP Effect Analysis**

SnpEff (v4.2) (Cingolani et al. 2012) was run for LUND1 using a gene annotation database tailored to the TB ancestor reference genome (Comas et al. 2010). With the exception of setting the upstream and downstream effect interval to 100 nucleotides, we used default settings. Variant positions unique to LUND1 in comparison to the L4 dataset alignment were filtered, and the SnpEff results for these positions, in addition to gene descriptions from the NCBI Gene database, can be found in Table S9 of Additional File 1.

Of note were two mce-associated genes thought to be involved in macrophage invasion (Gagneux 2018) that showed MODERATE or MODIFIER impact annotations: *mce1C* (missense variant) and *mce1B* (downstream gene variant). A MODERATE impact variant (missense variant) was also flagged in *zmp1*, which may be involved in host-pathogen interaction (Correa et al. 2014).

Additionally, there were numerous variant positions that had MODERATE or MODIFIER impact annotations in genes involved in cell wall or membrane functions, as determined through Mycobrowser (Kapopoulou et al. 2011): *mmpL4* (missense variant)*, mmpS4* (downstream gene variant)*,* Rv0102 (upstream gene variant), Rv0912 (downstream gene variant), *ctpG* (missense variant), Rv1999c (missense variant), *cysA1* (missense variant), *cysW* (missense variant), *lppR* (downstream gene variant), *merT* (missense variant), Rv3104c (downstream gene variant), Rv3273 (missense variant), Rv3635 (downstream gene variant), and Rv3821 (downstream gene variant).

**Birth-death model parameterization**

For both datasets, birth death multi-rho tree priors were utilized. The L4 birth death skyline analyses were parameterized as follows for both BDSKY+UCLD+origin and BDSKY+UCLD. The rho parameter, referencing the sampling proportion at each sampling time, was split into four dimensions. One dimension was provided for each ancient sample, with a Beta prior distribution with mean 0.01, and one dimension was provided for modern genome representation, with Beta prior distribution with mean 0.1. The rho sampling times for each ancient genome were given as their respective tip dates, and the rho sampling time for modern genomes was set to 0. For the MTBC dataset, the rho parameter had a prior mean estimate of 1.31E-2 (95% HPD interval: 4.75E-3, 0.0223) and a posterior mean estimate of 2.102E-3 (95% HPD interval: 7.23E-4, 3.7E-3). For the L4 dataset, the rho parameter had a prior mean estimate of 1.57E-2 (95% HPD interval: 3.5E-3, 0.0301) and a posterior mean estimate of 3.034E-3 (95%HPD interval: 6.54E-4, 5.85E-3). The reproductive number parameter, described here as the ratio between the diversification and extinction rate of lineages, was given 5 dimensions. The becomeUninfectiousRate was given one dimension for the ancient data with a log normal distribution with mean 0.001, and one dimension for the modern data with a log normal distribution with mean 0.1.

For the L4 BDSKY+UCLD+origin analysis we imposed an upper limit on the origin of 10,000 years, with a starting value of 4,000 years (the initial value of the origin must be greater than the initial tree height estimate), and a uniform distribution.

**Ratio of lineage diversification to lineage extinction**

For the data presented here as analyzed through the BDSKY+UCLD model, we can describe the parameter describing population dynamics as the ratio of lineage diversification to lineage extinction. For both datasets, we see an increase in this ratio at approximately 750 BP. In the MTBC model, it increases sharply and maintains its peak between 4 and 5. The increase is more gradual in the L4 model, and declines to hovering just above 1. This roughly coincides with a jump in effective population size estimated by Liu and colleagues for MTBC lineages indigenous to China (Liu et al. 2018). The decline of the diversification/extinction ratio for L4 beginning approximately 350 BP appears surprising, given the historically recorded rise of the White Plague in Europe from the 17^th^-19^th^ century (Dubos and Dubos 1952).

Prior estimates of R, the analog epidemiological parameter in outbreak settings, for populations of tuberculosis were restricted by geography or specific outbreak, and were dynamic within and between studies. Stadler (2011) and Kühnert and colleagues (2018) have used Bayesian phylodynamic approaches to estimate R for tuberculosis in the United States and in two regional outbreaks, respectively. Ma and colleagues reviewed R estimates from traditional epidemiology studies, and found estimates to range between 0.24 to 4.3 (Ma et al. 2018).

**
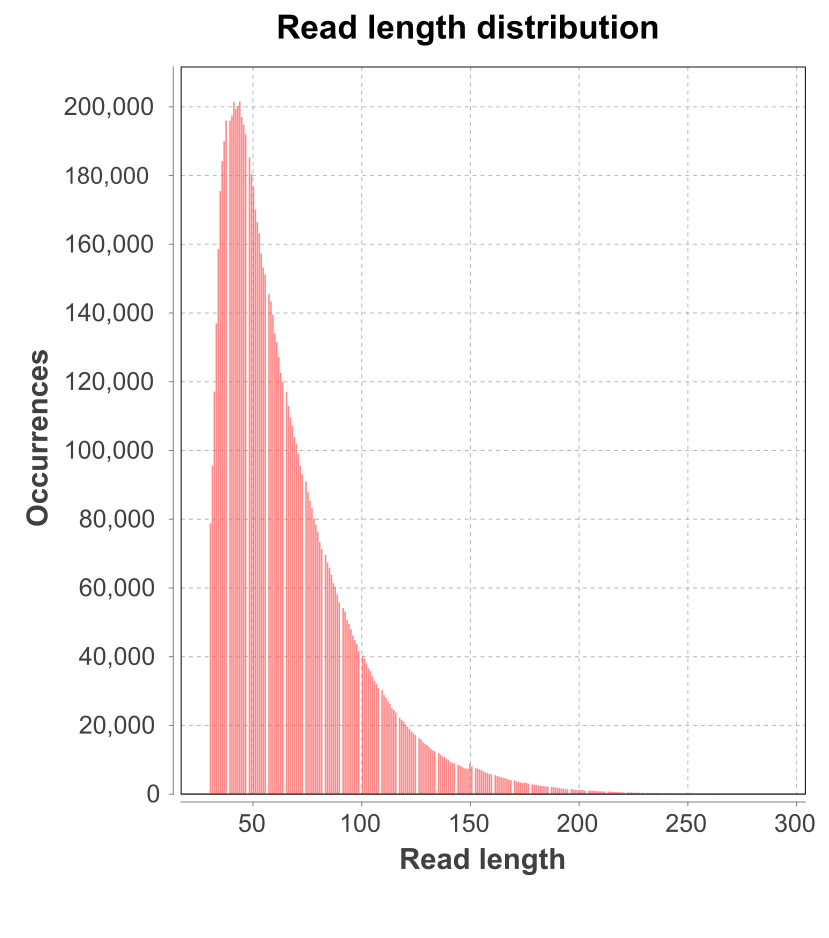
**

**Figure S1. Fragment length distribution plot for 150 cycle, paired-end sequencing data from UDG-treated, TB captured LUND1 library.** Fragment lengths as provided by DamageProfiler in the EAGER pipeline for 9,482,901 reads mapped to the TB ancestor genome with BWA as implemented in EAGER (-l 32, -n 0.1, -q 37).

**
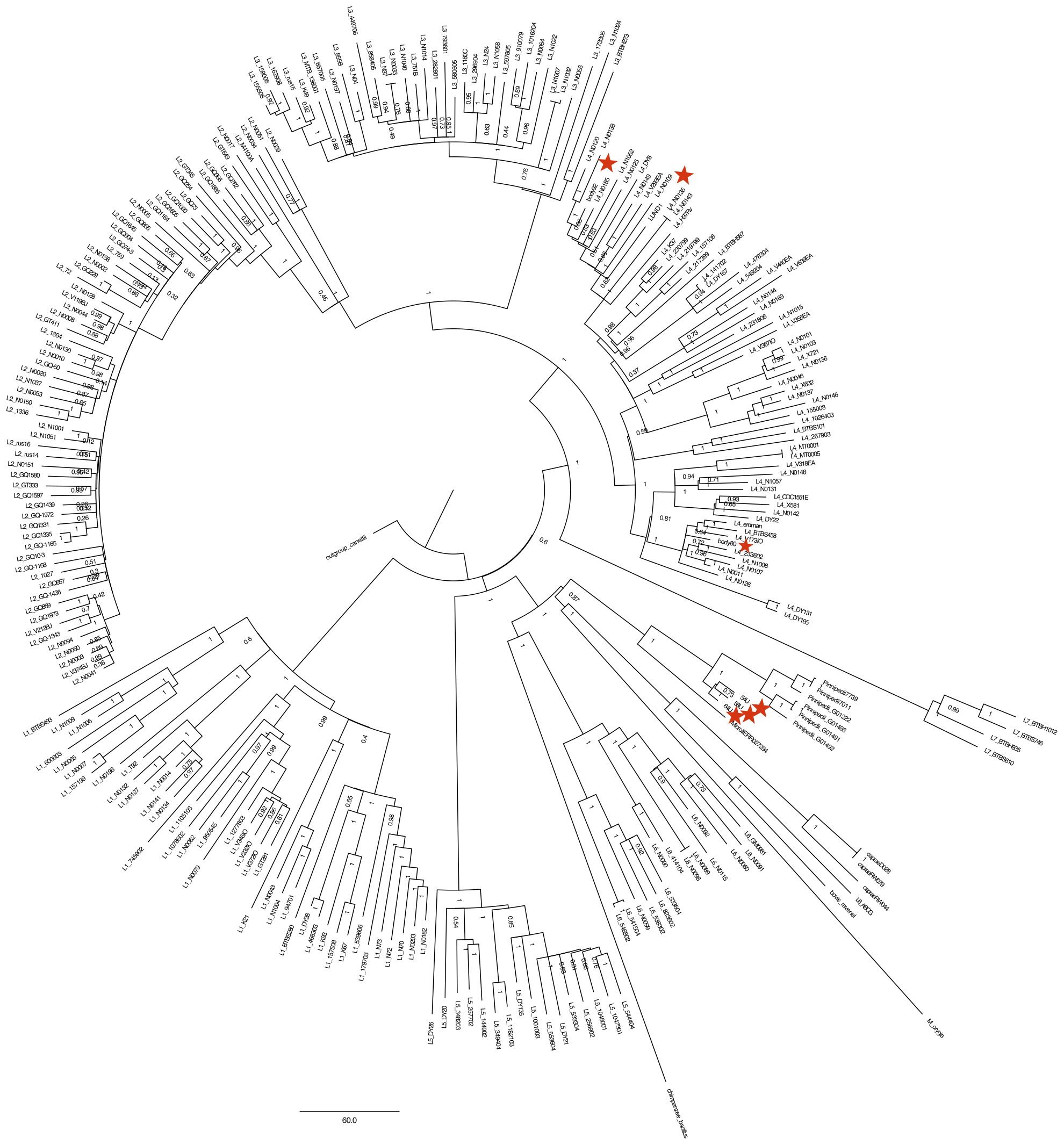
Figure S2. MTBC dataset neighbor joining tree.** The neighbor joining tree was configured using MEGA-Proto and generated with MEGA-CC, with 500 bootstrap replicates. The red stars indicate the ancient genomes.

**
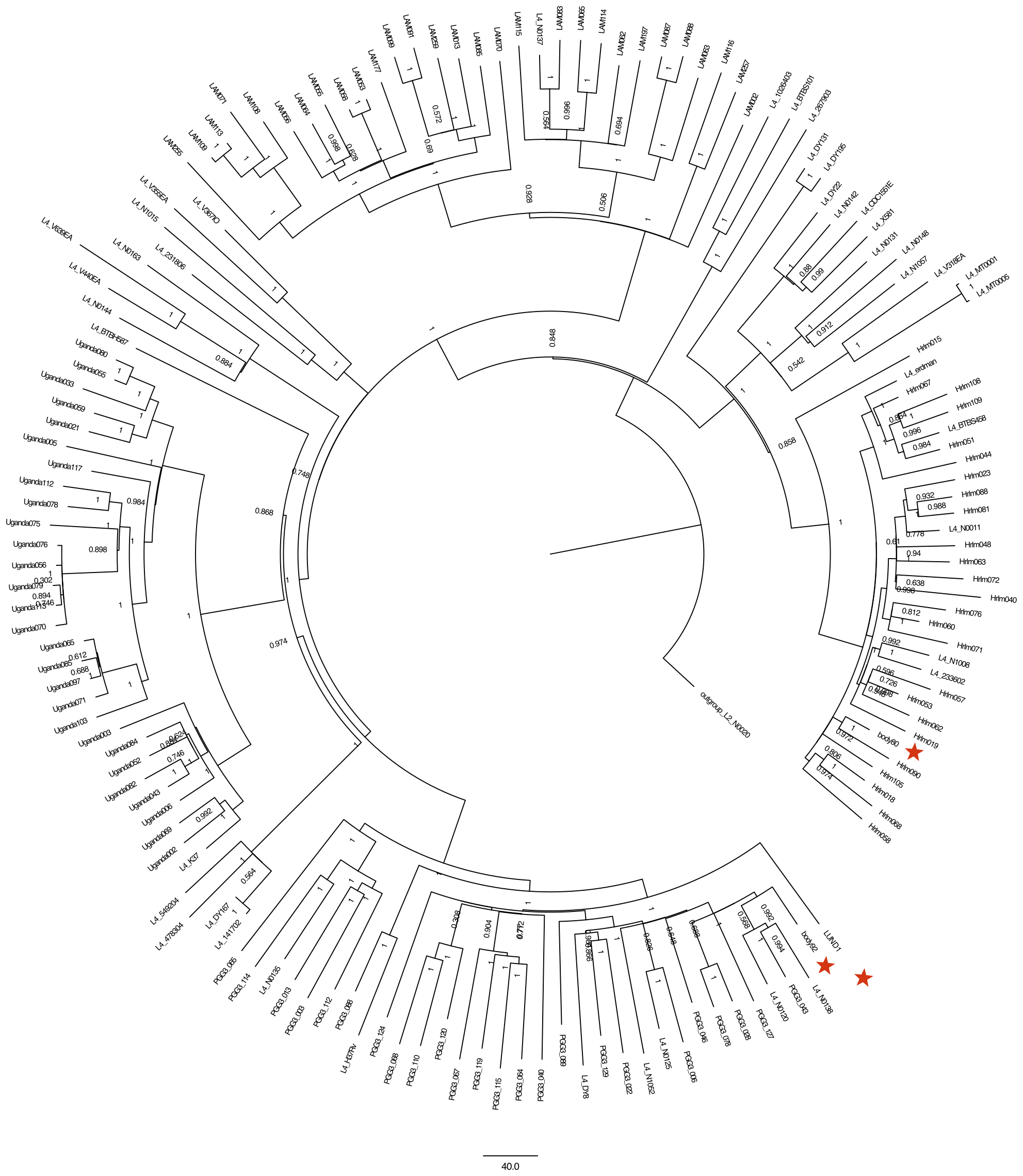
**

**Figure S3. L4 dataset neighbor joining tree.** The neighbor joining tree was configured using MEGA-Proto and generated with MEGA-CC, with 500 bootstrap replicates. The red stars indicate the ancient genomes.

**
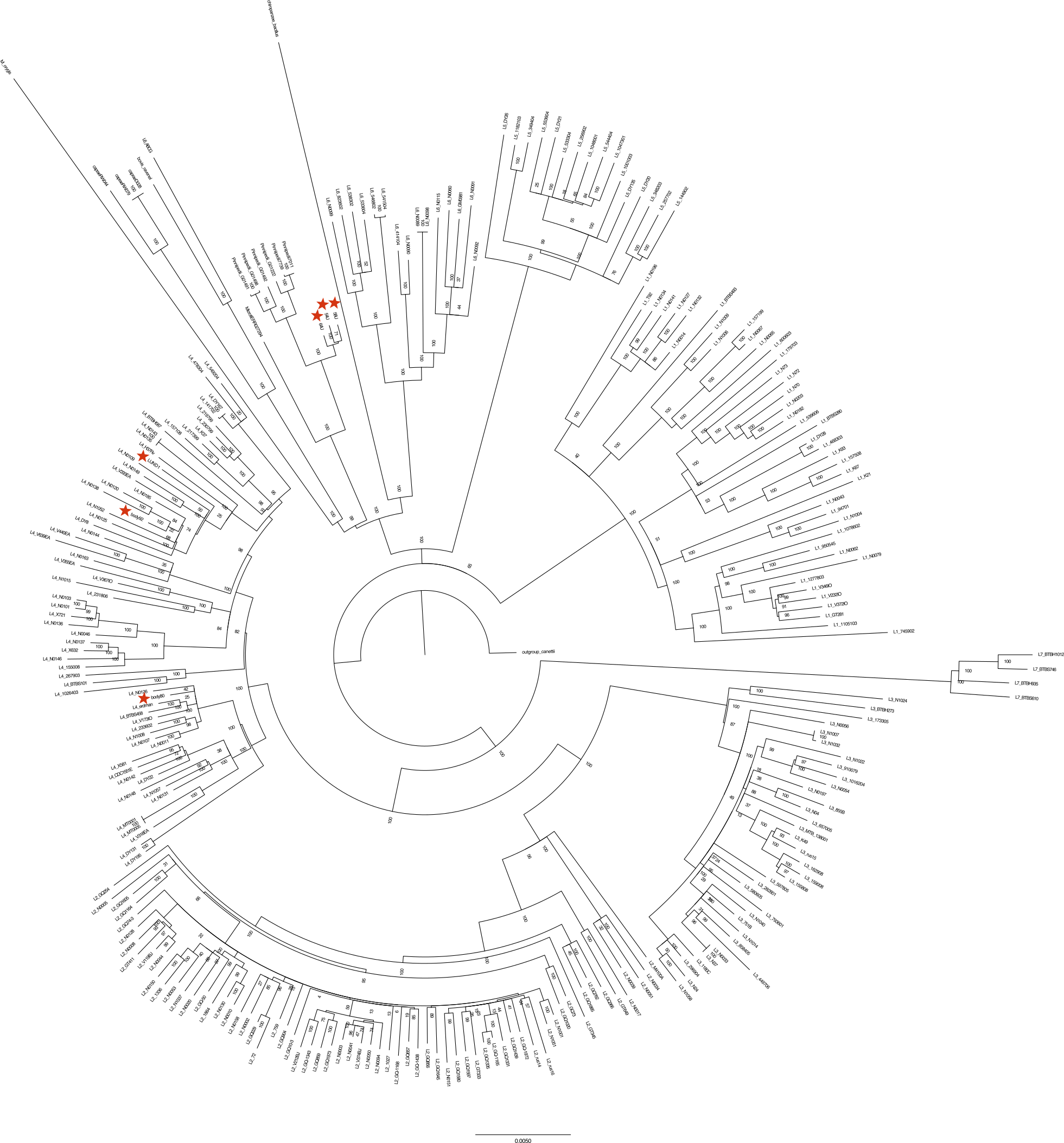
**

**Figure S4. MTBC dataset maximum likelihood tree.** The maximum likelihood tree was configured and generated with RAxML, with 500 bootstrap replicates. The red stars indicate the ancient genomes.

**
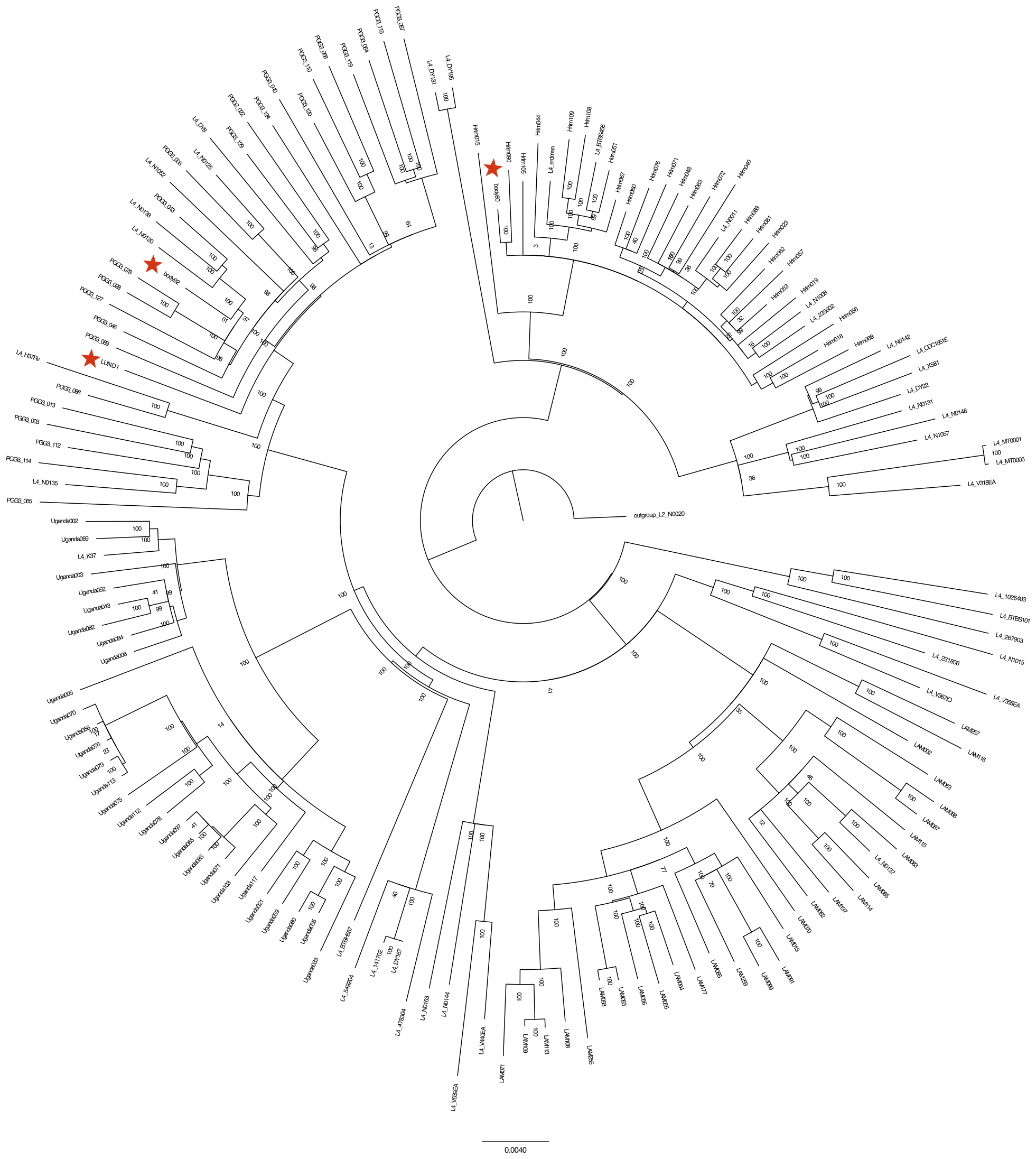
**

**Figure S5. L4 dataset maximum likelihood tree.** The maximum likelihood tree was configured and generated with RAxML, with 500 bootstrap replicates. The red stars indicate the ancient genomes.

**
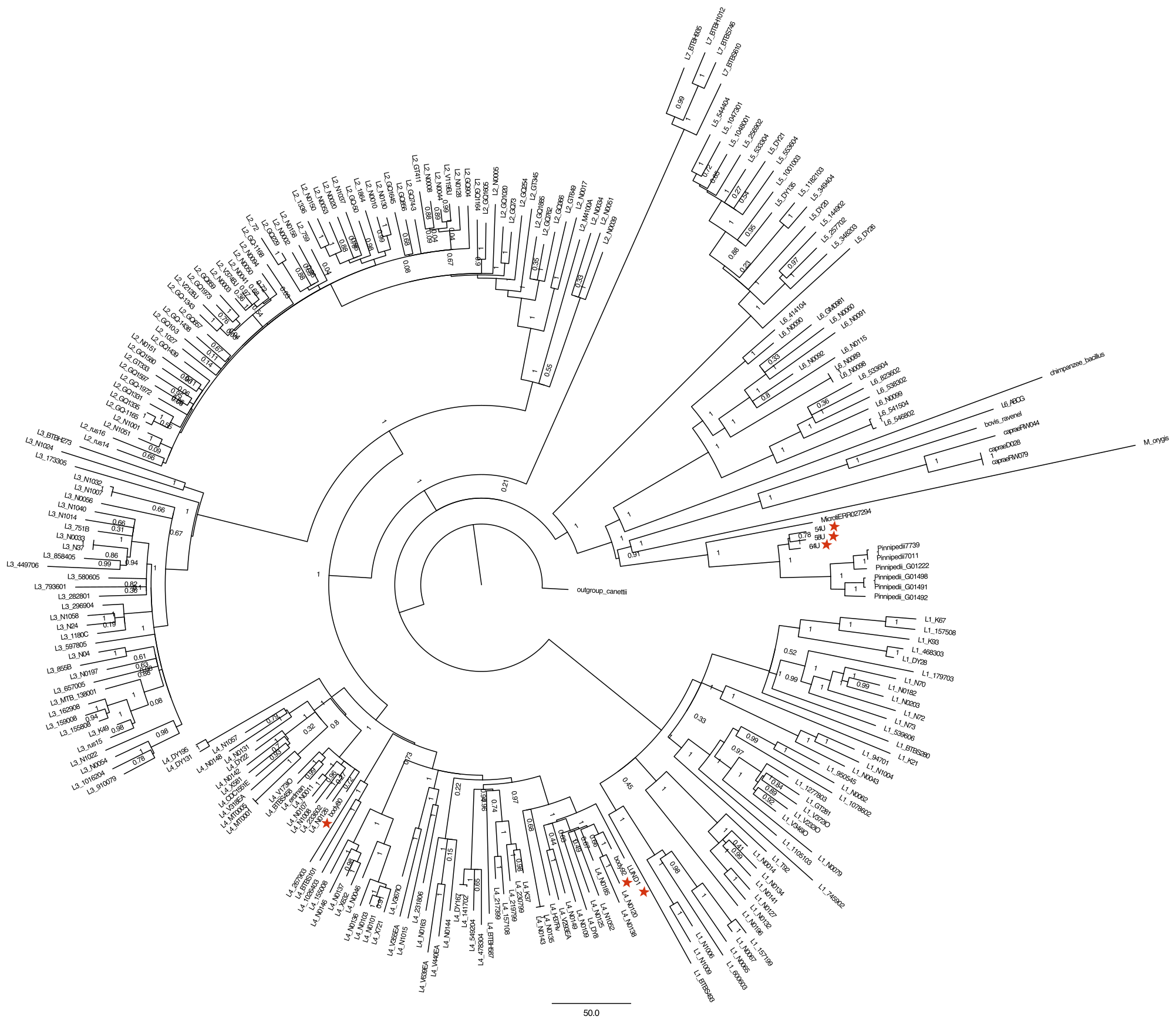
Figure S6. MTBC dataset maximum parsimony tree.** The maximum parsimony tree was configured using MEGA-Proto and generated with MEGA-CC, with 500 bootstrap replicates. The red stars indicate the ancient genomes.

**
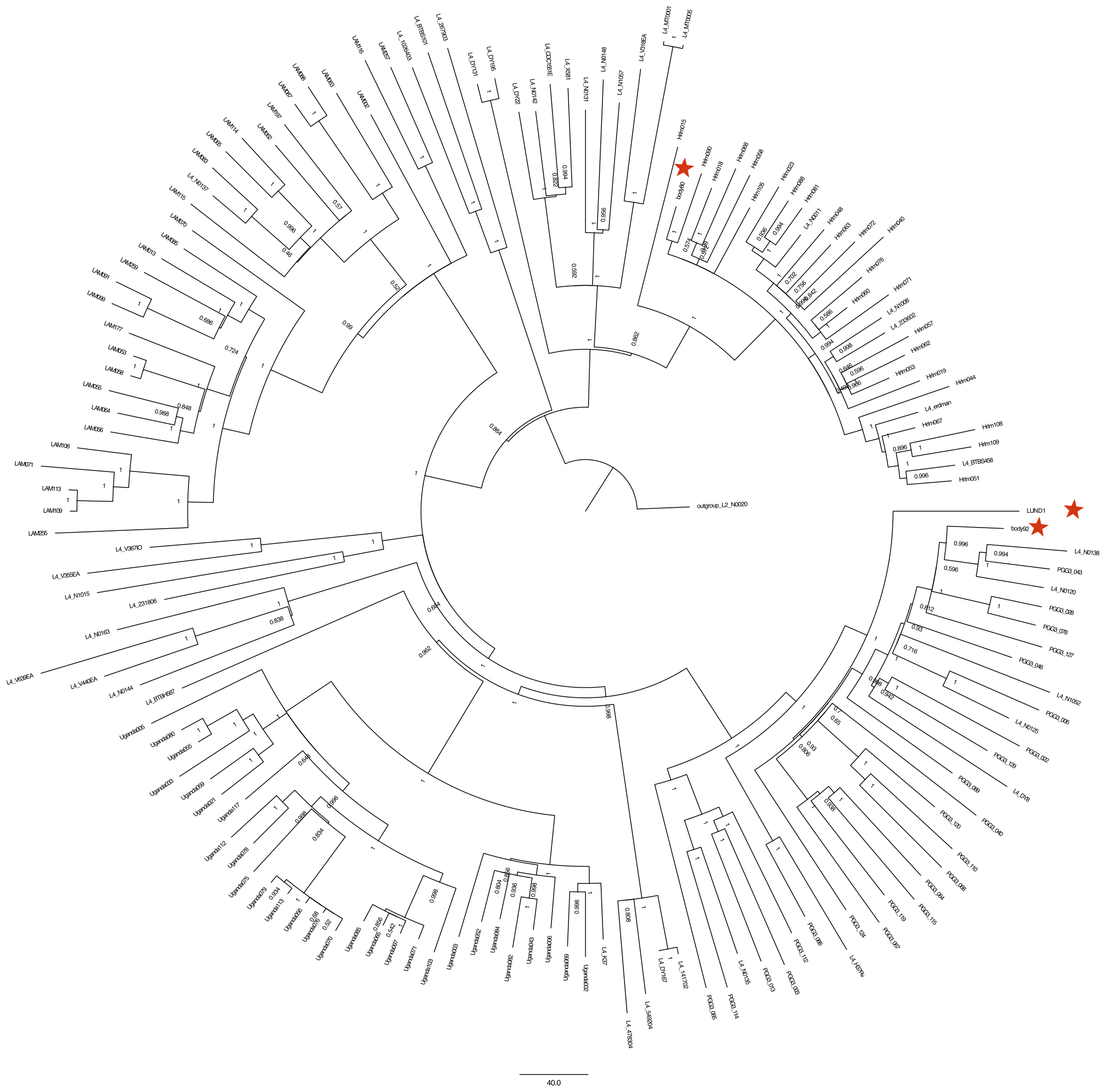
**

**Figure S7. L4 dataset maximum parsimony tree.** The maximum parsimony tree was configured using MEGA-Proto and generated with MEGA-CC, with 500 bootstrap replicates. The red stars indicate the ancient genomes.


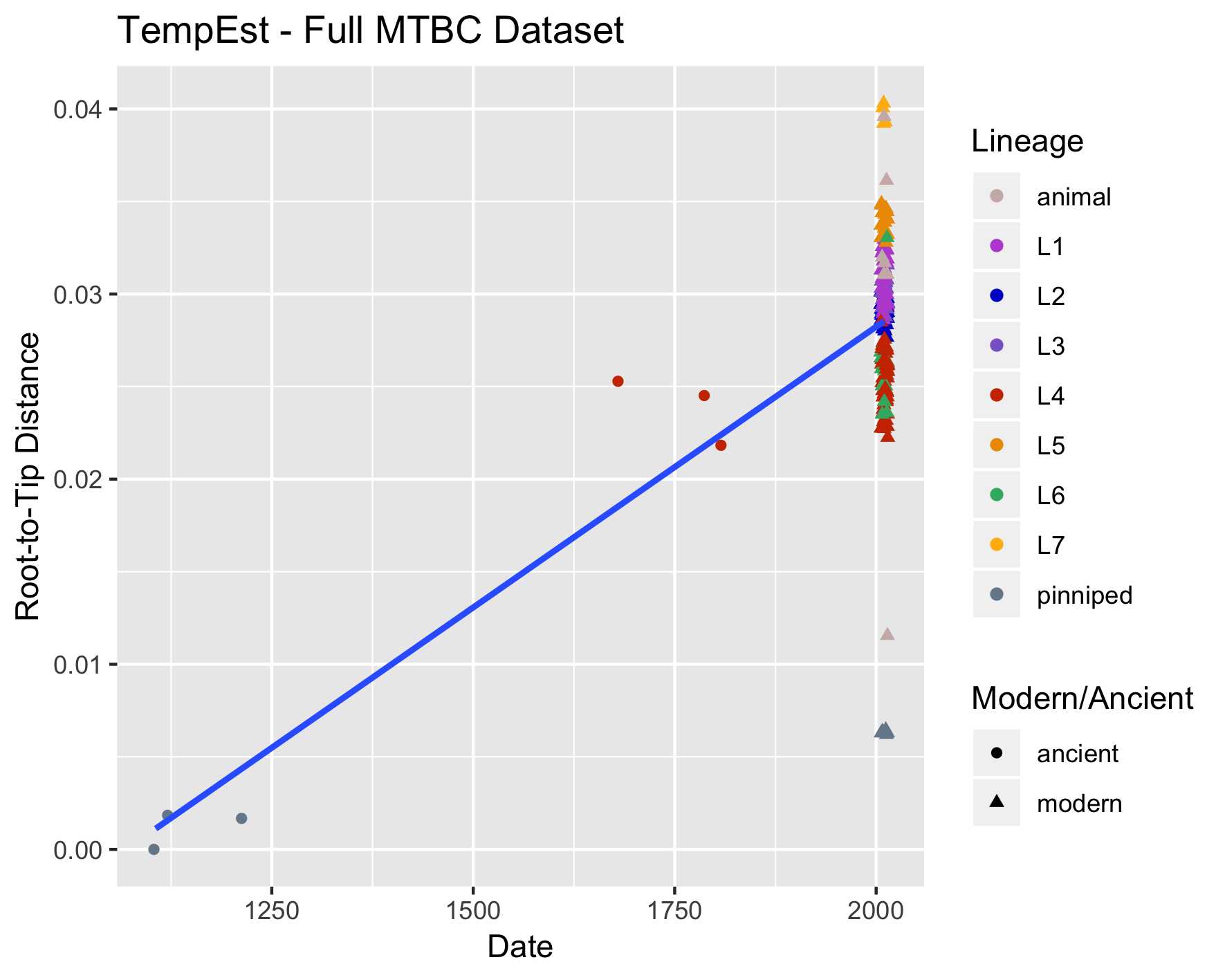


**Figure S8. TempEst plot for full MTBC dataset.** Plot generated from root-to-tip distances calculated in TempEst. R^2^ = 0.27.


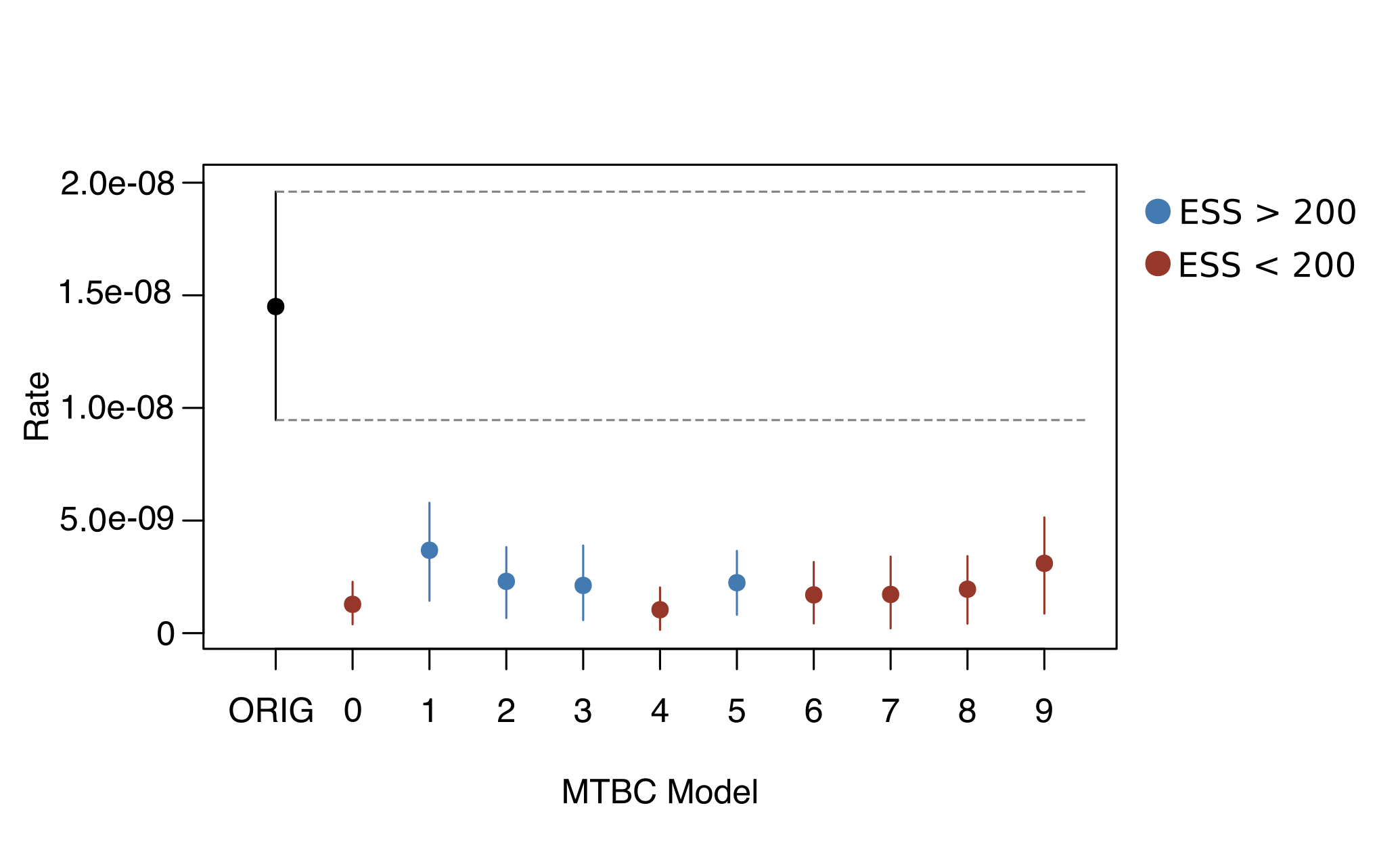


**Figure S9. Substitution rate comparison across date randomizations for the BDSKY+UCLD model of the MTBC dataset.** The circle represents the mean value for the rate parameter and the whiskers represent the 95% HPD interval of the rate parameter. “ORIG” refers to the original model with true tip dates. Among the randomized models, the circle and whiskers are colored according to rate parameter ESS range. The dotted line represents the upper and lower 95% HPD interval boundaries for the original model. None of the randomized models have 95% HPD intervals that overlap with this range.


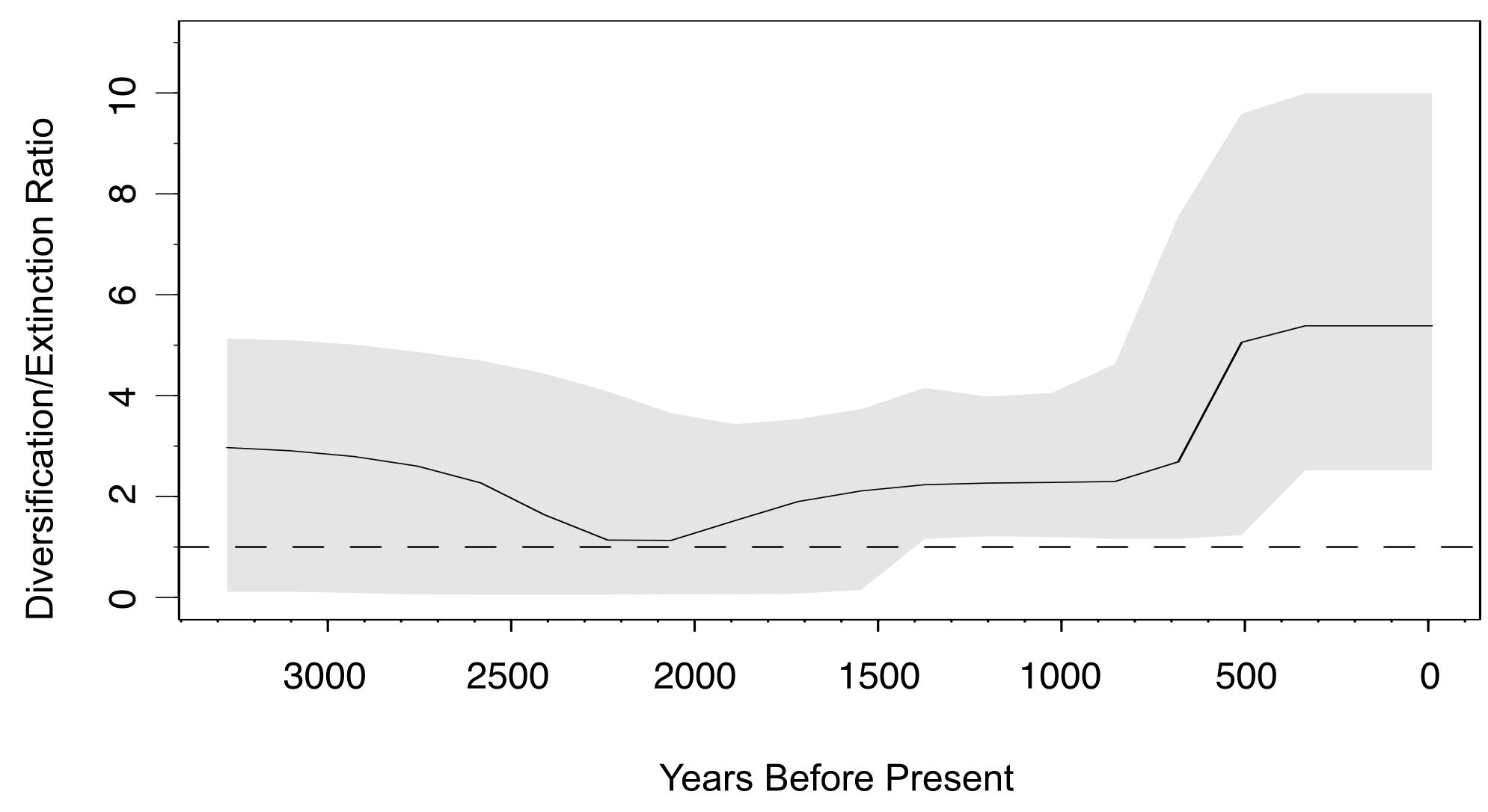


**Figure S10. Full MTBC BDSKY plot.** The central black line indicates mean ratio of lineage diversification to lineage extinction over time, the shaded grey area represents the 95% HPD interval of ratio over time, and the dashed line indicates the ratio is equal to 1.


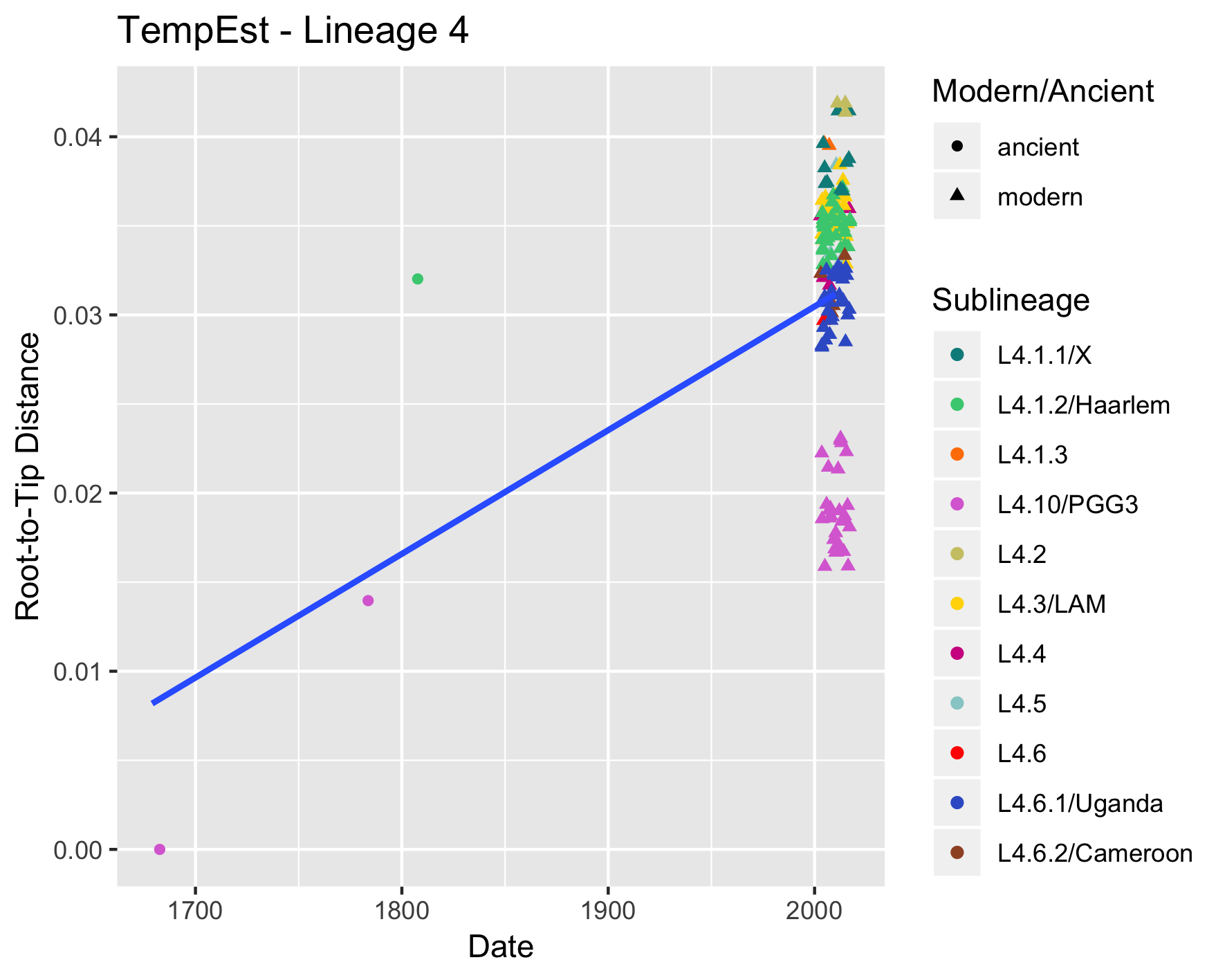
 **Figure S11. TempEst plot for Lineage 4 dataset.** Plot generated from root-to-tip distances calculated in TempEst. R^2^ = 0.11.


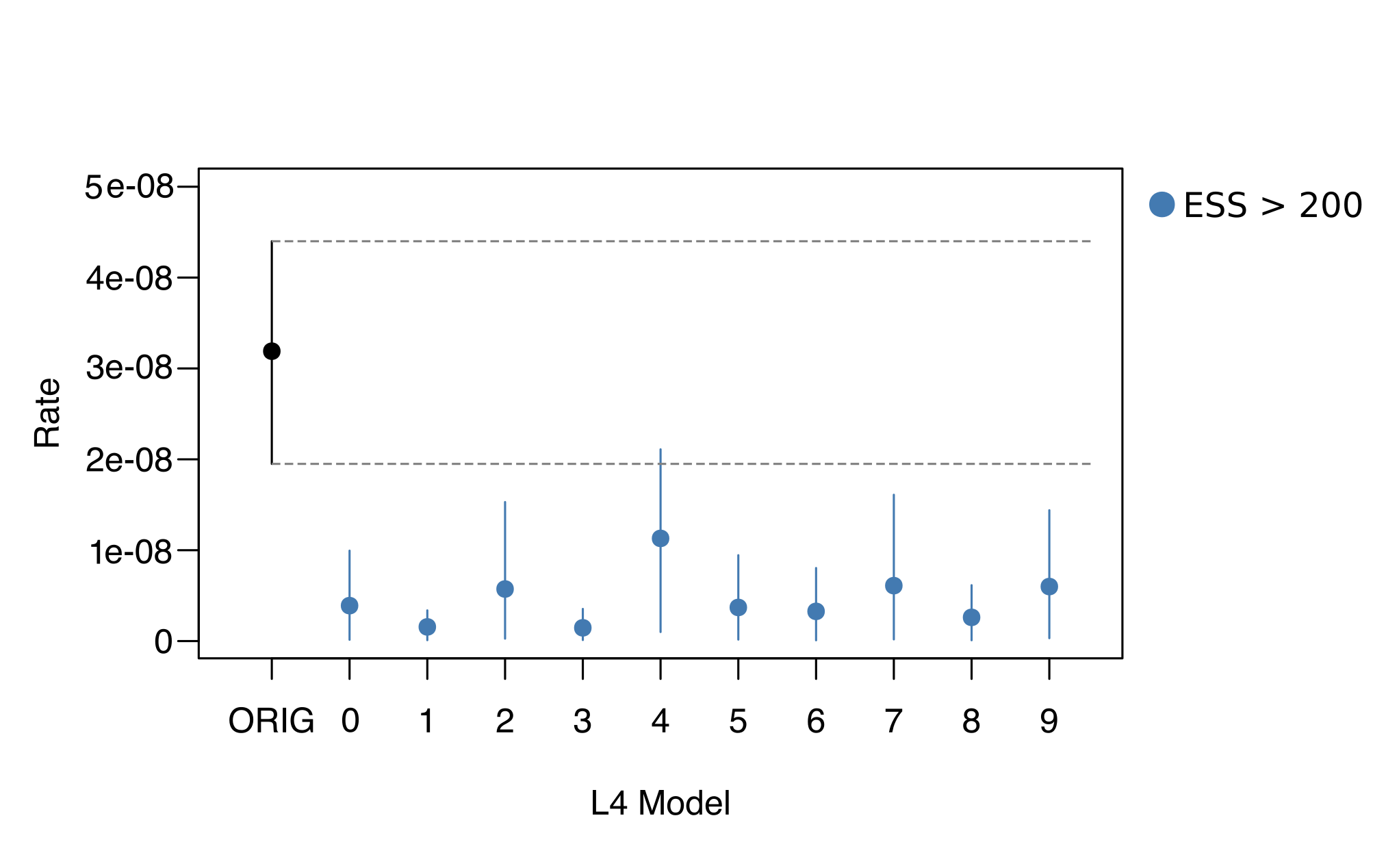


**Figure S12. Substitution rate comparison across date randomizations for the BDSKY+UCLD model of the L4 dataset.** The circle represents the mean value for the rate parameter and the whiskers represent the 95% HPD interval of the rate parameter. “ORIG” refers to the original model with true tip dates. Among the randomized models, the circle and whiskers are colored according to rate parameter ESS range. The dotted line represents the upper and lower 95% HPD interval boundaries for the original model. Nine out of ten randomized models (all apart from replicate 4) have 95% HPD intervals that overlap with this range.


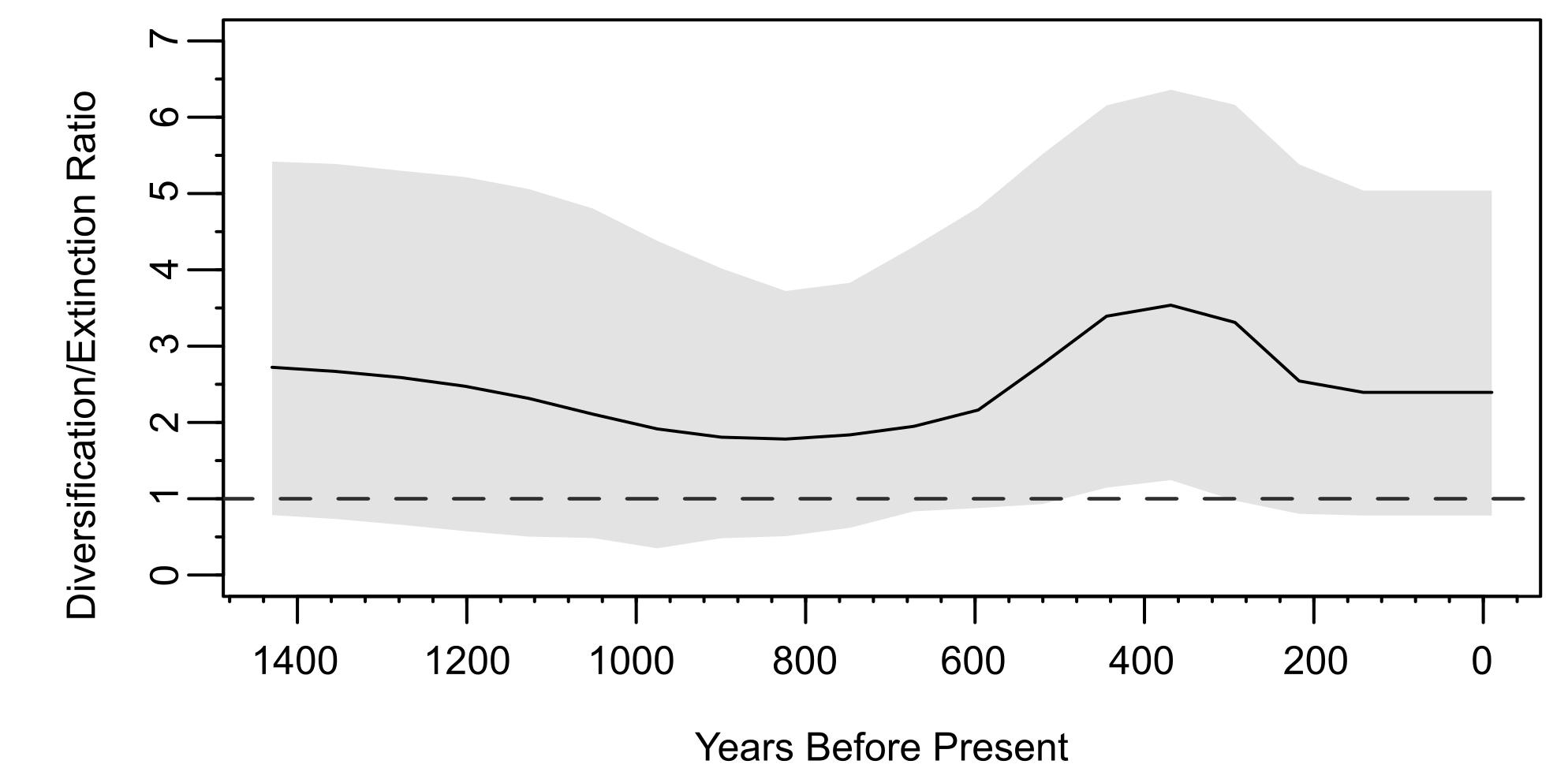


**Figure S13. L4 BDSKY plot.** Birth-death skyline plot from the BDSKY+UCLD+noOrigin model applied to the L4 dataset. The central black line indicates mean ratio of lineage diversification to lineage extinction over time, the shaded grey area represents the 95% HPD interval of ratio over time, and the dashed line indicates the ratio is equal to 1.

**
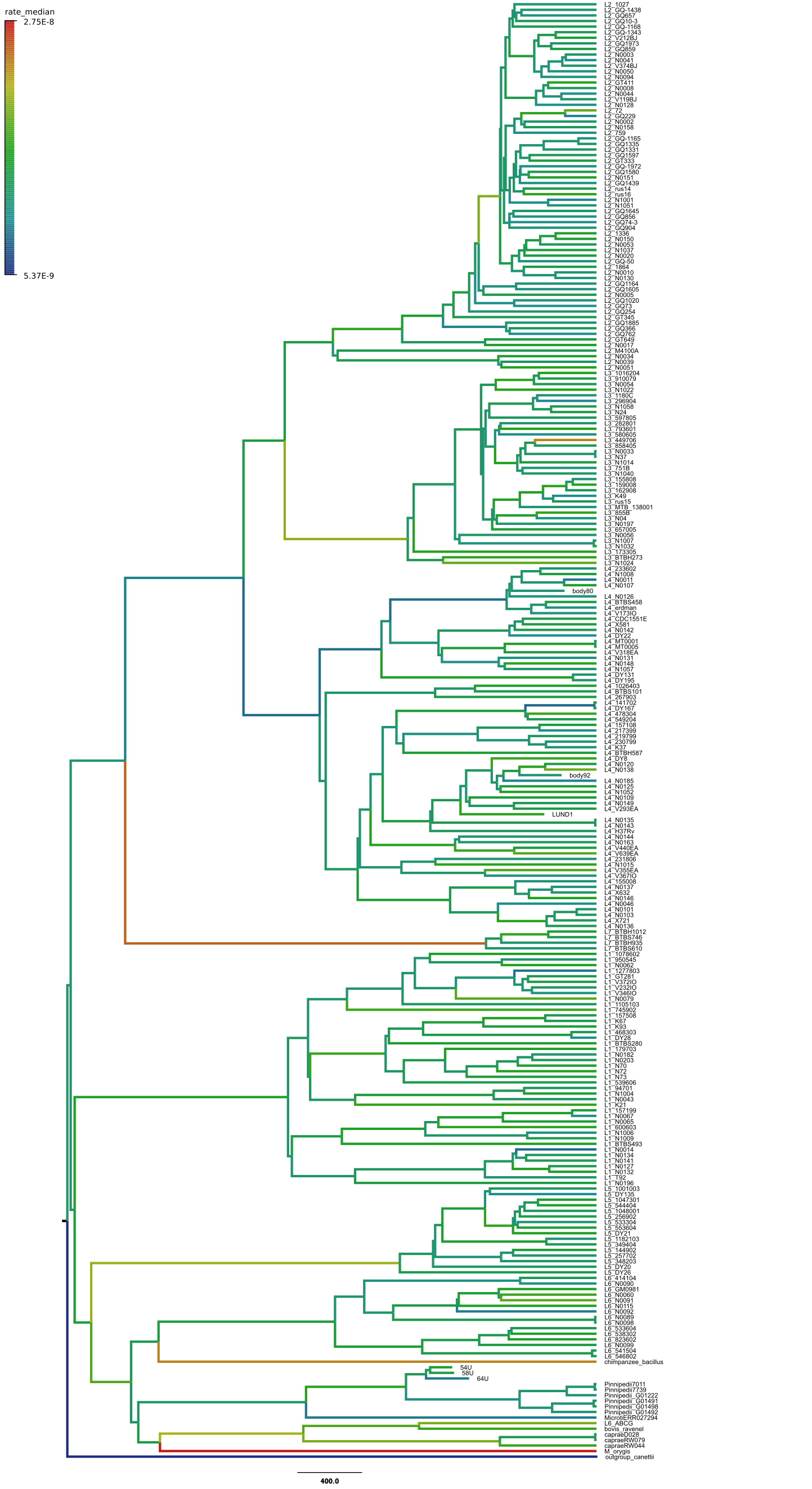
**

**Figure S14. MTBC BDSKY+UCLD maximum clade credibility tree.** Branches of the tree were colored according to median substitution rate.

**
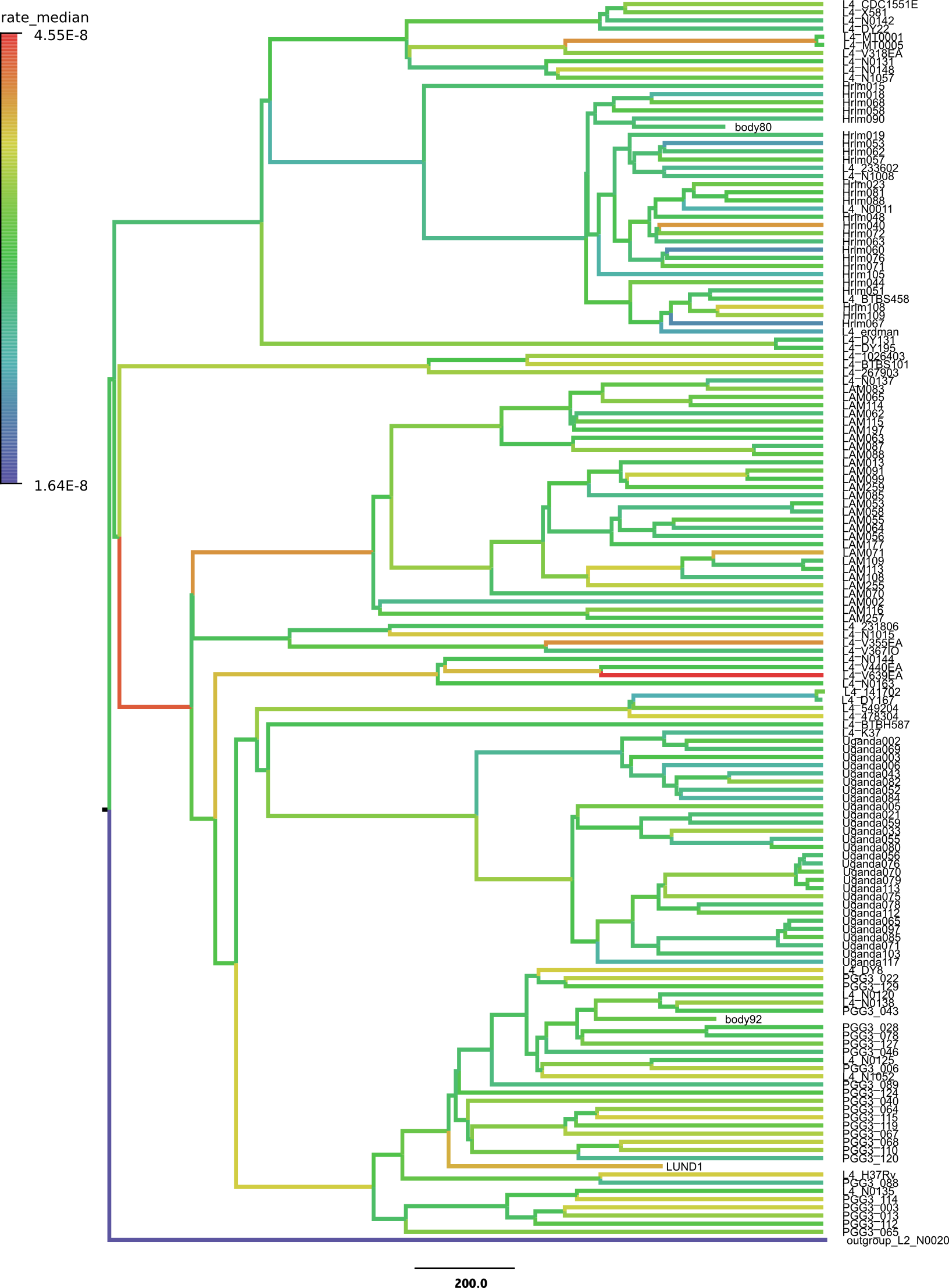
**

**Figure S15. L4 BDSKY+UCLD maximum clade credibility tree.** Branches of the tree were colored according to median substitution rate.


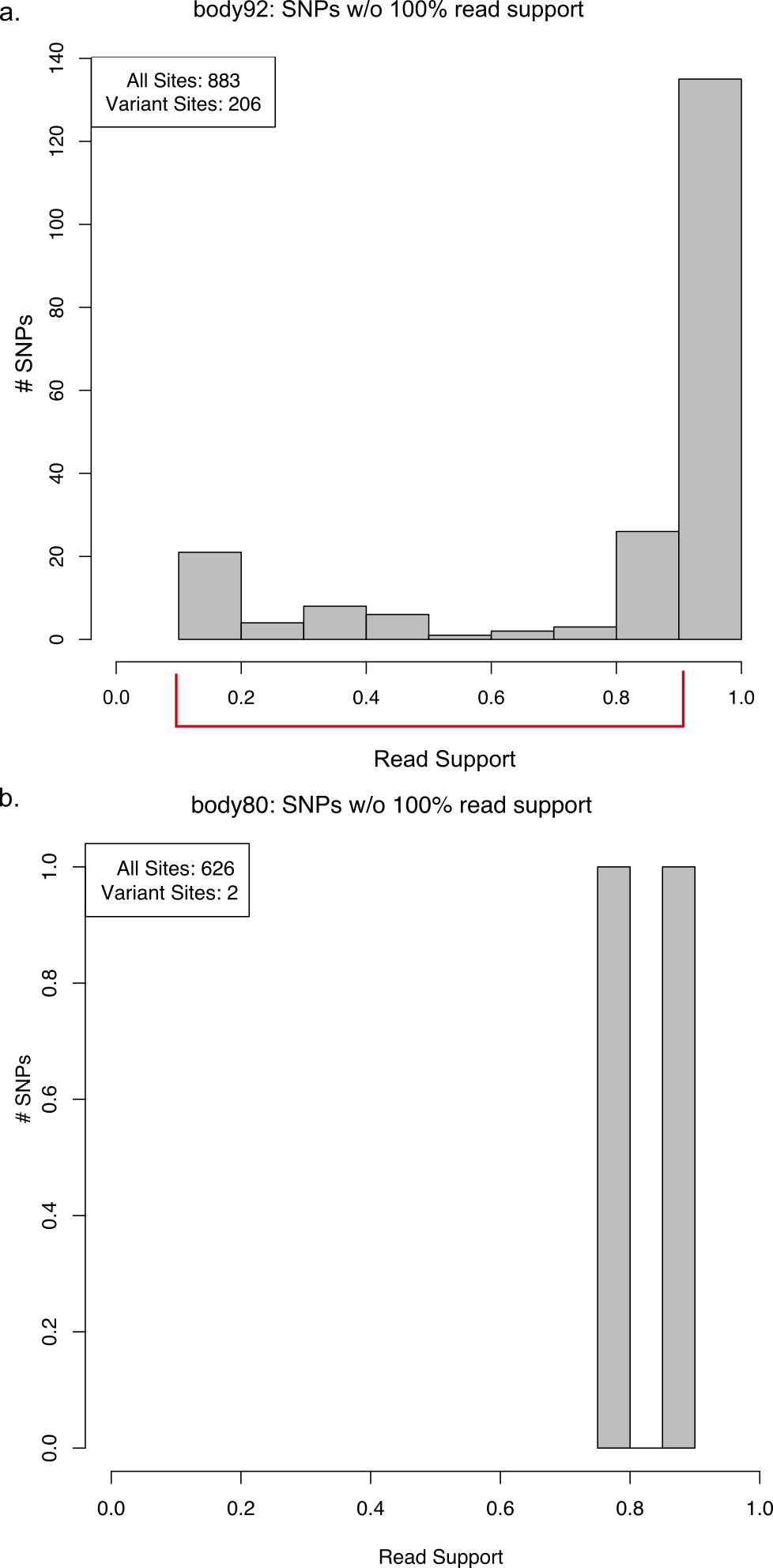


**Figure S16. Heterozygosity plots for body92 and body80.** A) The grey bars represent the quantity of sites at which between 10-99% of the reads represent an alternative derived allele (the allele must have coverage of 5-fold or greater to be counted). Body92 has 206 of these sites in total, though most of them have >90% representation, making them dominant alleles. The minority alleles, falling between 10-90% of the reads representing a given site (within the red bracket), are 70 in total. B) The grey bars represent the quantity of sites at which between 10-99% of the reads represent an alternative derived allele. Body80 has only two variant positions.

**
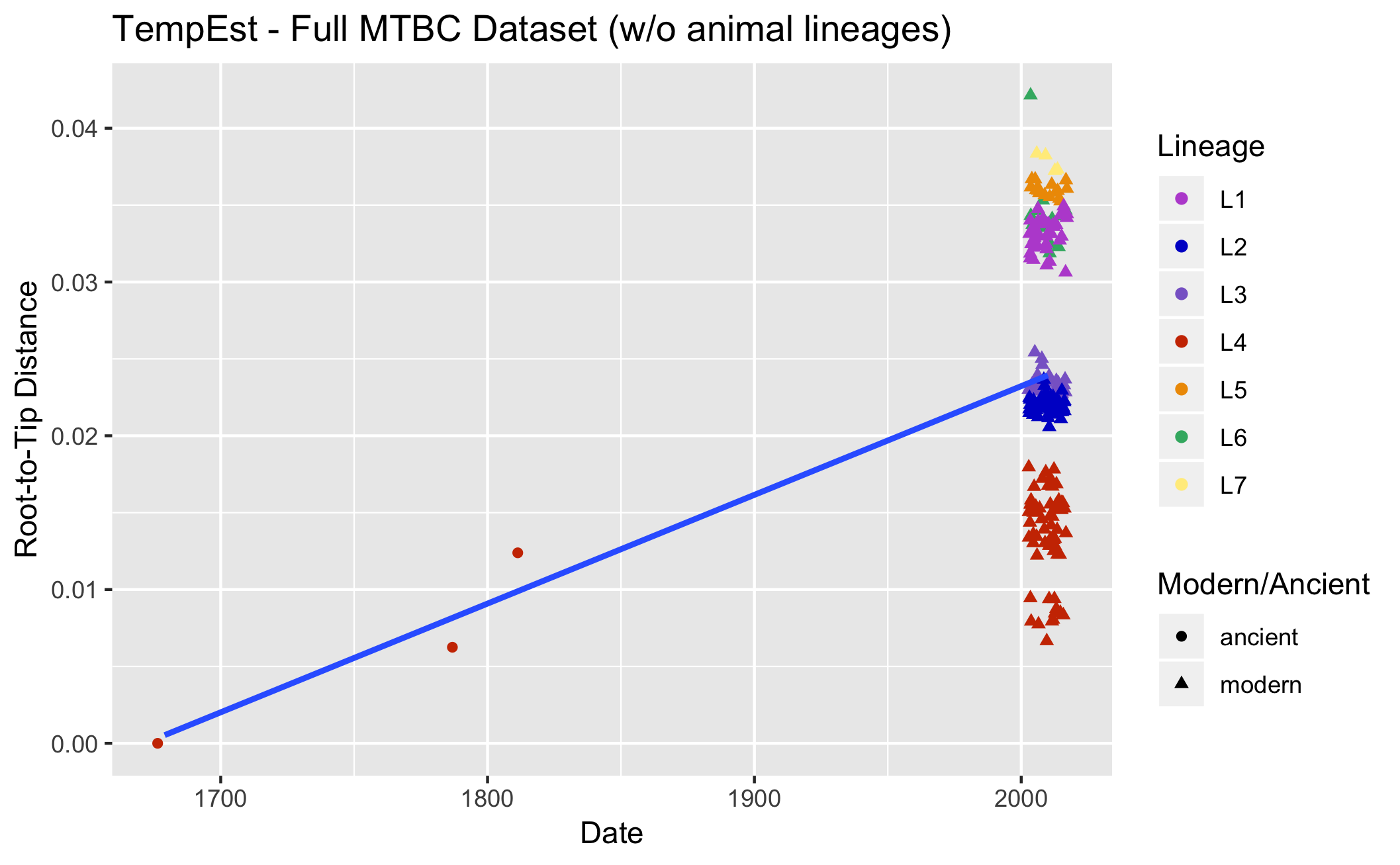
**

**Figure S17. TempEst plot for full MTBC excluding animal lineages.** Plot generated from root-to-tip distances calculated in TempEst. Input was a maximum likelihood tree generated with RAxML based on a SNP alignment excluding animal lineages of the MTBC, and consequently excluding the ancient *M. pinnipedii* genomes. R^2^ = 0.06.
