# Supplementary material for: A seventeenth-century Mycobacterium tuberculosis genome supports a Neolithic emergence of the Mycobacterium tuberculosis complex
Source: Genome Biol. 2020 Aug 10;21:201. doi: 10.1186/s13059-020-02112-1 (PMC7418204; doi:10.1186/s13059-020-02112-1)
Supplement: Supplementary file 12 — Additional file 12. Review history. [file 13059_2020_2112_MOESM12_ESM.docx]

Review History

**First round of review**

**Reviewer 1**

**Are you able to assess all statistics in the manuscript, including the appropriateness of statistical tests used?**

Yes, and I have assessed the statistics in my report.

**Comments to author:**

This well written manuscript presents aDNA analyses of a calcified nodule dissected from the mummified remains of a Swedish Bishop who died in 1697. The ancient DNA obtained from the remains is of high quality, with an average genome-wide coverage of ~140X after enrichment. The aDNA extraction, bioinformatics and phylogenetic analyses are carefully done. Results confirm those of other aDNA studies of Mtb and are consistent with Mtb evolutionary rate estimates using diverse methods. These results place the TMRCA of globally extant Mtb within 6,000 years.

Specific comments:

Line 207. The authors refer to Boritsch 2016, to support the idea that the clonal evolution of Mtb is “a point of contention.” Boritsch et al experimentally demonstrated LGT via distributive conjugal transfer in Mycobacterium canettii. They did not demonstrate LGT in Mtb. LGT is obvious in genomic data from M. canettii. There is abundant evidence of clonal evolution/ complete linkage of sites of Mtb, and no convincing evidence of LGT. This is not a point of contention in the field.

Line 231. Data are plural.

Figure 3. The line indicating R0 = 1 is hard to see.

Line 320-334. Discussion of archeological evidence of TB from >6,000 years BP. The authors discuss rate variation as a possible explanation of the discrepancy between their results and archeological studies. While rate variation is an important concept to raise, this study and others (i.e. Duchene 2016) have shown that there is very little evidence of rate variation in Mtb. This contrasts with other bacterial pathogens e.g. Yersinia pestis. There are simpler and more relevant explanations for the discrepancy which I think should be discussed here. First, the osteological traces of TB are not specific. For example, other pathogens can cause chronic vertebral osteomyelitis (e.g. dimorphic fungi, which can also cause calcific lung lesions as discussed viz Histoplasmosis in the manuscript). On what basis was TB identified in the archeological studies cited here? I recommend a more thorough discussion of these data.

Second, the TMRCA of the extant global population of Mtb is not equivalent to the timing of emergence of TB. Ancient populations of pathogenic mycobacteria may have existed prior to the emergence of what we now recognize as MTBC. Indeed, the study of Gutierrez, which is cited here, suggests MTBC arose from diverse, well mixed progenitor populations of pathogenic mycobacteria. Sabin et al point out later that M canettii causes a disease indistinguishable from tuberculosis (Lines 435-). By parsimony, the MRCA of MTBC and M. canettii would have been virulent. Numerous species among the Mycobacteria are pathogenic to humans and, in fact, an even more distantly related species (M kansasii) causes tuberculosis-like lung disease. However, even if this were not the case, it would be quite possible for MTBC to have arisen from ancient pathogen populations that left no other traces in modern populations.

Line 346-7. The sentence starting “Despite the ambiguity” makes no sense. The finding of a European and African origin for L4 in different studies does not contradict the idea of historically close contacts between the two regions. It is supportive evidence of historically close ties between these regions, as discussed in both of the studies cited here.

Lines 347-. Discussion of Stucki et al. Stucki et al’s classification of “specialist” and “generalist” sub-lineages of L4 is nothing more than a label applied to sub-lineages with different levels of geographic dispersion. The authors go on to speculate that “an ancestral strain underwent an evolutionary event in which some descendent lineages acquired or lost a feature that equipped them to expand past limited host niches into Eurasia.” This speculation is unjustified – first, because it neglects the impact of neutral phenomena on Mtb evolution. As an obligate human pathogen Mtb is entirely dependent on humans to migrate and human migration is a complex behavior that has and continues to be shaped by numerous influences that have nothing to do with tuberculosis. There are many studies describing the neutral phenomena that have shaped Mtb migration. These studies should be discussed here. Second, there is no basis for describing humans in Africa as a “limited” niche for Mtb. From the perspective of human genetic diversity, environments within Europeans are limited with respect to African populations and of course the African continent comprises diverse environments, climates, cultures, societies etc. An equally if not more likely explanation is that the L4 sub-lineages were historically associated with human sub-populations with different levels of mobility that resulted in variable patterns of dispersion observed now.

Lines 378-. It would be beneficial to include a discussion of previously published estimates of R0 for TB (i.e. from epidemiological modeling studies) to contextualize the finding of historical variation in lineage birth/death rates presented here.

Line 385- This sentence is confusing. I think the authors mean that more comprehensive sampling, including closely related strains, would be required to resolve recent temporal fluctuations in R0. This needs to be clarified. The paper would also benefit from a greater depth of discussion of the disparity between these findings and those presented in Liu et al. How do the samples and/or analyses differ?

Line 403- While calcification is often thought of as a feature of longstanding infection, it can occur during acute infection. This sentence is inaccurate.

Line 406- It is quite disingenuous to state that the pathogen preservation described in Kay 2014 and Devault 2017 “rivals” that described in this manuscript. Kay et al obtained 6-fold coverage of a Brucella melitensis genome from shotgun sequencing data, whereas Devault et al obtained 299-fold coverage from shotgun data. The pre-capture coverage of the Mtb genome presented here is 0.018x. As assessed by the quantity and quality of ancient pathogen DNA, the tissues studied by Kay et al and Devault et al were better preserved than the material examined here.

Line 440- A bottleneck is a neutral phenomenon. If you wish to refer to an adaptive process, “selective sweep” or “clonal replacement” are more accurate descriptors. Neutral and adaptive phenomena are both plausible explanations.

Line 451- The sentence beginning “However, their membership…” is unjustifiably presented as a fact. We don’t know what evolutionary pressures were experienced by historical populations of M. pinnipedii-like organisms.

**Reviewer 2**

**Are you able to assess all statistics in the manuscript, including the appropriateness of statistical tests used?**

Yes, and I have assessed the statistics in my report.

**Comments to author:**

Review of GBIO-D-19-00166 entitled "A seventeenth-century Mycobacterium tuberculosis genome supports a Neolithic emergence of the Mycobacterium tuberculosis complex" by Sabin et al.

This paper describes the reconstruction of an ancient MTBC genome isolated from a TB nodule found within the chest cavity of Bishop - Peder Winstrup of Lund. The genome was obtained post detection of MTBC reads from an analysis of shotgun data. The authors follow up with a whole genome enrichment and manage to reconstruct a very nice high quality TB genome- one of the most convincing to date - which they then use to try and date the origin of not only the MTBC complex but, perhaps a bit more convincingly lineage L4.

I have no complaints about the data generation and curation nor the draft-genome 'reconstruction'. These are all top notch. I do have several problems with the analysis and over interpretation of the genome.

Currently there are only a handful of verified ancient TB genomes - the one presented here - and a more complicated set from a Hungarian mummy - with possible co-infection - which the authors attempt to reconstruct denovo for their analyses here. The additional genomes - don't fall within the diversity of human MTBC strains, but clearly represent a novel emergence from some unknown source (some of the authors have published on these separately) but fall today- on an incompletely sampled diversity- nearest to seals and Pinniped TB. The paper attempts to use these ancient tip dates (as heavily stressed in the discussion) as serial sampled data points to bypass the issues of time dependency, but then relies on not just the two European strains - but rather the ancient Peruvian ones as well -which likely represent a novel jump from some unknown species, and are therefore unrelated to the current circulating MTBC global strains and likely have very different evolutionary rates is senseless. I don't believe the data at hand can answer the question they ask about MTBC origins.

The authors go on to estimate R- to do this they need to know the proportion of the total number of TB cases that they have sampled. It is completely unclear how this proportion was obtained? It is unclear how the birth-death model can adequately address this without proper priors.

Skyline approaches like the ones used here only make sense when the sequences are sampled from a totally panmictic population. The data presented here are clearly not panmictic and thus any spatial subdivision undermines the analysis. Have the authors considered this in their analysis and if so how have they accounted for it?

The authors don't show their a linear regression (please include) anywhere - but cite values that are really poor, particulary for L4 (R2 = 0.113). It's hard to imagine there's a real clock like signal on those data at all. This would suggest that the clock only explains 11% of the total variance!

In addition, the credible intervals on the skyline plots are so large that it would possible to draw a straight line through the plot at a value of R = 3 on the first one and R =2 on the second one. How can that be possibly excluded. I believe the authors are placing far too much value on the population estimates (or R) generated from these plots.

In summary, I believe the MTBC reconstruction is novel and solid work, but the molecular clock analysis, skyline plots and estimates of R are completely overdrawn and should be tempered heavily.

**Authors Response**

**Point-by-point responses to the reviewers’ comments:**

**Reviewer #1:**

**Comment 1:** Line 207. The authors refer to Boritsch 2016, to support the idea that the clonal evolution of Mtb is “a point of contention.” Boritsch et al experimentally demonstrated LGT via distributive conjugal transfer in Mycobacterium canettii. They did not demonstrate LGT in Mtb. LGT is obvious in genomic data from M. canettii. There is abundant evidence of clonal evolution/ complete linkage of sites of Mtb, and no convincing evidence of LGT. This is not a point of contention in the field.

*We agree with Reviewer #1 on the significance of Boritsch et al. 2016, and we now see that the placement of the citation in the original manuscript was misleading. We have re-worded the sentence in question to be clearer and more direct (lines 207-210).*

**Comment 2:** Line 231. Data are plural.

*Thank you, we have fixed this typo.*

**Comment 3:** Figure 3. The line indicating R0 = 1 is hard to see.

*In this revision of the manuscript, we have reduced our focus on the birth-death skyline plots in the main text and shifted the plots themselves to the Supplementary Information in Additional File 2 (Fig. S10 and S13). For both plots, we have substituted the grey line indicating R=1 to a black perforated line.*

**Comment 4.1:** Line 320-334. Discussion of archeological evidence of TB from >6,000 years BP. The authors discuss rate variation as a possible explanation of the discrepancy between their results and archeological studies. While rate variation is an important concept to raise, this study and others (i.e. Duchene 2016) have shown that there is very little evidence of rate variation in Mtb. This contrasts with other bacterial pathogens e.g. Yersinia pestis. There are simpler and more relevant explanations for the discrepancy which I think should be discussed here.

*While it is true there has been little evidence of rate variation in the MTBC, and we ourselves found only a very small degree of rate variation between lineages in our analyses, we agree with Reviewer 1 that it is an important concept to discuss in any molecular dating paper. Given the still-sparse representation of Mycobacterium tuberculosis complex genomes through time, we would like to retain our discussion of rate variation as one possible explanation for the disagreement between genetic and other lines of evidence. We have included a sentence acknowledging our finding of very little rate variation, while also*

*reinforcing the importance of these findings being based on temporally structured populations (lines 348-356).*

**Comment 4.2:** First, the osteological traces of TB are not specific. For example, other pathogens can cause chronic vertebral osteomyelitis (e.g. dimorphic fungi, which can also cause calcific lung lesions as discussed viz Histoplasmosis in the manuscript). On what basis was TB identified in the archeological studies cited here? I recommend a more thorough discussion of these data.

*We agree it is possible the discrepancy between our dates for the emergence of the MTBC*

*and the archaeological evidence could be due to the misdiagnosis of skeletal lesions, and now stress in our manuscript that diagnoses from skeletal analyses are interpretations of evidence and are not necessarily evidence in and of themselves (lines 339-342). However, a review of the resolution in TB diagnosis offered through gross analysis of archaeological material is outside the focus of the current manuscript. We prefer to mention misdiagnosis as a possibility, and give equal weight to it as we do to other theoretical reasons for the discrepancy.*

**Comment 4.3:** Second, the TMRCA of the extant global population of Mtb is not equivalent to the timing of emergence of TB. Ancient populations of pathogenic mycobacteria may have existed prior to the emergence of what we now recognize as MTBC. Indeed, the study of Gutierrez, which is cited here, suggests MTBC arose from diverse, well mixed progenitor populations of pathogenic mycobacteria. Sabin et al point out later that M canettii causes a disease indistinguishable from tuberculosis (Lines 435-). By parsimony, the MRCA of MTBC and M. canettii would have been virulent. Numerous species among the Mycobacteria are pathogenic to humans and, in fact, an even more distantly related species (M kansasii) causes tuberculosis-like lung disease. However, even if this were not the case, it would be

quite possible for MTBC to have arisen from ancient pathogen populations that left no other traces in modern populations.

*We agree with Reviewer 1, and address this point in the Discussion section amidst the limitations of our study (see lines 448-455). We have expanded this paragraph to be clearer and included a citation of Gutierrez et al. 2005 in the section discussing possible pathogenic diversity prior to the event that gave rise to the extant MTBC (line 452-455).*

**Comment 5:** Line 346-7. The sentence starting “Despite the ambiguity” makes no sense. The finding of a European and African origin for L4 in different studies does not contradict the idea of historically close contacts between the two regions. It is supportive evidence of historically close ties between these regions, as discussed in both of the studies cited here.

*We thank Reviewer 1 for pointing this out and have removed the phrase “Despite the ambiguity” from the beginning of the sentence in question.*

**Comment 6.1:** Lines 347-. Discussion of Stucki et al. Stucki et al’s classification of “specialist” and “generalist” sub-lineages of L4 is nothing more than a label applied to sublineages with different levels of geographic dispersion. The authors go on to speculate that “an ancestral strain underwent an evolutionary event in which some descendent lineages acquired or lost a feature that equipped them to expand past limited host niches into Eurasia.” This speculation is unjustified – first, because it neglects the impact of neutral phenomena on Mtb evolution. As an obligate human pathogen Mtb is entirely dependent on humans to migrate and human migration is a complex behavior that has and continues to be shaped by numerous influences that have nothing to do with tuberculosis. There are many studies describing the neutral phenomena that have shaped Mtb migration. These studies should be discussed here.

*We agree with Reviewer 1 and acknowledge the “specialist” and “generalist” classifications put forward by Stucki et al. are mere labels describing geographic distribution. We have adjusted the wording of this section to make this clearer (lines 369-372). It was not our intention to dismiss the potential role of neutral phenomena in this portion of the manuscript. Instead, we intended to suggest that investigating the ancestral node of the “specialist” L4.6 sublineage and “generalist” L4.10 sublineage could help elucidate whether this localization phenomenon, which is strongly suggested by the existing data, is a genetic phenomenon and not purely an artifact of human population movements or epidemiological sampling, and if so, by what variation it is driven. We are not asserting it absolutely must have a genetic basis, but given the existing evidence that L4 diversity in Africa was driven by European colonial migrations into the continent, it is curious (a) that some sublineages are global while some are localized and (b) that we do not see any sublineages localized to areas in Europe. We wonder why there seems to be a directionality to this phenomenon. We have removed the speculative statement quoted in Reviewer 1’s comment above and rephrase our point as a suggestion and question (lines 377-382). We also include an acknowledgment of genetic drift as a possible reason for this signal (lines 377-380).*

**Comment 6.2:** Second, there is no basis for describing humans in Africa as a “limited” niche for Mtb. From the perspective of human genetic diversity, environments within Europeans are limited with respect to African populations and of course the African continent comprises diverse environments, climates, cultures, societies etc. An equally if not more likely explanation is that the L4 sub-lineages were historically associated with human subpopulations with different levels of mobility that resulted in variable patterns of dispersion observed now.

*We agree that Africa is not ecologically or epidemiologically limiting for the MTBC and recognize Africa holds the greatest diversity of MTBC lineages. In this sentence we meant that the “specialist”, or highly localized (non-globally distributed) sublineages of L4 as defined by Stucki and colleagues all occur on the African continent. We have altered the wording of the sentence to be clearer and to avoid the suggestion that the ecology of Africa is somehow limiting for the organisms (lines 369-373).*

**Comment 7:** Lines 378-. It would be beneficial to include a discussion of previously published estimates of R0 for TB (i.e. from epidemiological modeling studies) to contextualize the finding of historical variation in lineage birth/death rates presented here.

*After revisions to the manuscript, we have removed the bulk of discussion of the R0 value from the main text to the Supplementary Information in Additional File 2. In this analysis, we have included a brief reference to the two studies we are aware of that presented R calculated through Bayesian phylodynamic methodologies, and a review paper discussing estimates of R from traditional epidemiological studies (Additional File 2, lines 81-109).*

**Comment 8:** Line 385- This sentence is confusing. I think the authors mean that more comprehensive sampling, including closely related strains, would be required to resolve recent temporal fluctuations in R0. This needs to be clarified. The paper would also benefit from a greater depth of discussion of the disparity between these findings and those presented in Liu et al. How do the samples and/or analyses differ?

*We have removed the sentence in question. Upon a re-visitation of the text, we found it misleading. We consider our sampling of L4 to be representative of available diversity. However, our dataset does not result from a single outbreak, and thus we can only infer R as a measure of secondary lineage birth rather than secondary infection birth. On a related note, after revisions to the manuscript, we have shifted most discussion of R to the Supplementary Information in Additional File 2.*

**Comment 9:** Line 403- While calcification is often thought of as a feature of longstanding infection, it can occur during acute infection. This sentence is inaccurate.

*Though acute infections can result in nodule formation, we argue that such nodules occur rarely, are usually diffuse, and form outside the chest. The imaging of Bishop Winstrup’s remains revealed a Ranke complex, which is a well-established sign of chronic tuberculosis infection. One co-author (Gracijela Bozovic), has 15 years of experience in radiology and has not seen calcification in the chest as the result of an acute infection. We have included references to support our argument in the main text at lines 108-114.*

**Comment 10:** Line 406- It is quite disingenuous to state that the pathogen preservation described in Kay 2014 and Devault 2017 “rivals” that described in this manuscript. Kay et al obtained 6-fold coverage of a Brucella melitensis genome from shotgun sequencing data, whereas Devault et al obtained 299-fold coverage from shotgun data. The pre-capture coverage of the Mtb genome presented here is 0.018x. As assessed by the quantity and quality of ancient pathogen DNA, the tissues studied by Kay et al and Devault et al were better preserved than the material examined here.

*We recognize our prior terminology as having been misleading, and have adjusted our phrasing.*

**Comment 11:** Line 440- A bottleneck is a neutral phenomenon. If you wish to refer to an adaptive process, “selective sweep” or “clonal replacement” are more accurate descriptors. Neutral and adaptive phenomena are both plausible explanations.

*We have included “selective sweep” in addition to “evolutionary bottleneck” in the sentence in question to leave open the possibilities of both neutral and adaptive phenomena (lines 452-455).*

**Comment 12:** Line 451- The sentence beginning “However, their membership…” is unjustifiably presented as a fact. We don’t know what evolutionary pressures were experienced by historical populations of M. pinnipedii-like organisms.

*We agree with Reviewer 1, and have altered the phrasing of this sentence to acknowledge our uncertainty regarding the evolutionary history of M. pinnipedii-like organisms (lines 465- 469).*

**Reviewer #2:**

This paper describes the reconstruction of an ancient MTBC genome isolated from a TB nodule found within the chest cavity of Bishop - Peder Winstrup of Lund. The genome was obtained post detection of MTBC reads from an analysis of shotgun data. The authors follow up with a whole genome enrichment and manage to reconstruct a very nice high quality TB genome- one of the most convincing to date - which they then use to try and date the origin of not only the MTBC complex but, perhaps a bit more convincingly lineage L4. I have no complaints about the data generation and curation nor the draft-genome 'reconstruction'. These are all top notch. I do have several problems with the analysis and over interpretation of the genome.

**Comment 1:** Currently there are only a handful of verified ancient TB genomes - the one presented here - and a more complicated set from a Hungarian mummy - with possible coinfection - which the authors attempt to reconstruct denovo for their analyses here. The additional genomes - don't fall within the diversity of human MTBC strains, but clearly represent a novel emergence from some unknown source (some of the authors have published on these separately) but fall today- on an incompletely sampled diversity- nearest to seals and Pinniped TB. The paper attempts to use these ancient tip dates (as heavily stressed in the discussion) as serial sampled data points to bypass the issues of time dependency, but then relies on not just the two European strains - but rather the ancient Peruvian ones as well -which likely represent a novel jump from some unknown species, and are therefore unrelated to the current circulating MTBC global strains and likely have very different evolutionary rates is senseless. I don't believe the data at hand can answer the question they ask about MTBC origins.

*We agree with Reviewer #2 that the ancient Mycobacterium pinnipedii genomes from Peru lie within a sparsely-sampled clade of the MTBC, and that the evolutionary dynamics of the animal-associated MTBC lineages are largely unknown. This would potentially impede dating analyses by calibrating the MTBC tree with genomes that represent a different evolutionary rate than the majority of extant human-infecting MTBC diversity. However, the birth-death skyline + relaxed clock model we draw upon in our analysis accommodates the possibility of different parts of the tree having different evolutionary rates. All the models featured in our manuscript, excluding the constant coalescent + strict clock model, accommodate interlineage variation. We believe the joining of LUND1 and the two Hungarian genomes (all of which fall within the human-associated Lineage 4) with the ancient Peruvian genomes (a) mitigates the possibility of the M. pinnipedii genomes driving rate estimates across the tree as the only ancient representatives, and (b) provides a stronger dating estimate for the full MTBC than a model using only the Lineage 4 genomes or only the Peruvian genomes due to increased representation across the complex. It is true that more thorough sampling of the modern animal-associated MTBC lineages and more diverse ancient genomes would (and will, in the future) provide a stronger dataset. However, we believe that our manuscript offers the best answer possible at present to the questions we ask about the timing of MTBC*

*emergence. We have now addressed this point in lines 465-476.*

**Comment 2:** The authors go on to estimate R- to do this they need to know the proportion of the total number of TB cases that they have sampled. It is completely unclear how this proportion was obtained? It is unclear how the birth-death model can adequately address this without proper priors.

*We thank the reviewer for pointing out that the presentation of an R estimate is misleading here. Since our dataset does not stem from a single (connected) outbreak, we cannot infer a reproduction number representing TB transmission dynamics. Instead, we obtain an estimate of the diversification-extinction ratio through time. We have renamed the parameter accordingly and shifted most of the related discussion to the Supplementary Information in Additional File 2.*

*Given the scarcity of available ancient MTBC genomes, it is currently difficult to estimate the total global MTBC diversity during each sampling window. Our priors for the rho parameter were relatively arbitrary. We would stress, however, that the coalescent-based models that have been commonly applied to datasets including sparsely sampled ancient representatives in the past, assume random, uniform, very low sampling proportions over time. We argue that this assumption is less appropriate for our dataset than our use of a multi-rho birth-death skyline model, which explicitly models the markedly non-random sampling process through time. As noted in Additional File 2, we set a rho prior beta distribution with a mean of 0.01 for each ancient sampling point in both datasets and a beta distribution with a mean of 0.1 for the modern samples, because we expect to have sampled a higher proportion of the respective genetic diversity today than in the past.*

**Comment 3:** Skyline approaches like the ones used here only make sense when the sequences are sampled from a totally panmictic population. The data presented here are clearly not panmictic and thus any spatial subdivision undermines the analysis. Have the authors considered this in their analysis and if so how have they accounted for it?

*We thank the reviewer for pointing out this limitation of our study, the discussion of which we have added to the revised manuscript (lines 484-487). We believe that the assumption of panmixia is not problematic for our molecular dating analyses. While spatial subdivision can bias estimates of population dynamics, it is unlikely to have a significant effect on the estimate of the evolutionary rate and hence the molecular dating, which is at the focus of our study. Indeed, our estimate agrees with previous and concurrent work (Bos et al., 2014; Eldholm et al., 2016; Kay et al., 2015; Menardo et al., 2019). Due to the low evolutionary rate a robust phylogeographic study estimating the evolutionary rate simultaneously with the migratory process is unfortunately infeasible as it would be subject to serious identifiability issues. In fact, a recent summary of the geographical distribution of the MTBC by each lineage presented by O’Neill and colleagues (O’Neill et al., 2019), was based on a previously published evolutionary rate estimate (which was obtained assuming panmixia).*

**Comment 4:** The authors don't show their linear regression (please include) anywhere - but cite values that are really poor, particulary for L4 (R2 = 0.113). It's hard to imagine there's a real clock like signal on those data at all. This would suggest that the clock only explains 11% of the total variance!

*We have added linear regression plots representing the TempEst results in Additional File 2 (Figures S8 and S11). Though root-to-tip distance seems to be largely dependent on lineage in the case of the MTBC dataset and sublineage in the case of the L4 dataset, we argue there is a temporal signal. To validate the clock-like behavior of the datasets, we performed date randomizations of the birth-death skyline analysis. These were computationally intensive analyses, and the shuffling of tip dates slowed the convergence process, especially for the MTBC dataset. However, we reached rate parameter effective sampling size (ESS) values of over 100 for all ten full MTBC randomizations and ESS values over 200 for four out*

*of ten. For the MTBC dataset, all randomizations had mean rate estimates and 95% HPD intervals that did not overlap with the 95% HPD interval of the true model, fulfilling the stringent criteria for evaluating date randomizations. For L4, all randomizations reached convergence with rate ESS values of greater than 200. Of 10 randomizations, 9 yielded clock rate means and 95% HPD intervals well outside that of the correctly calibrated dataset. The 95% HPD interval of one randomization overlapped slightly with that of the original. Despite this slight overlap, we are convinced that there is a meaningful signal in our data. Please find the results of the date randomization in Tables S9 and S10 in Additional File 1 and Figures S9 and S12 in Additional File 2.*

**Comment 5:** In addition, the credible intervals on the skyline plots are so large that it would possible to draw a straight line through the plot at a value of R = 3 on the first one and R =2 on the second one. How can that be possibly excluded. I believe the authors are placing far too much value on the population estimates (or R) generated from these plots. In summary, I believe the MTBC reconstruction is novel and solid work, but the molecular clock analysis, skyline plots and estimates of R are completely overdrawn and should be tempered heavily.

*We have tempered our discussion of our findings for R by (a) altering our phrasing to describe our presented population dynamic parameter for the BDSKY+UCLD models as the ratio of lineage diversification to extinction, and (b) shifting the birth-death skyline plots displaying the ratio of lineage diversification and extinction and its 95% HPD interval for each dataset to Additional File 2 (Figures S10 and S13) along with the more in-depth discussion of the values we find (Additional File 2, lines 67-86).*

**Second round of review**

**Reviewer 1**

The paper has been substantially improved in response to reviewer comments.

A few minor issues:

Line 209-210 - the authors state that LGT occurs infrequently among M. canettii. This is incorrect. Experimental conjugation is infrequently observed in laboratory settings (per Boritsch, which is cited here). Natural populations show evidence of frequent recombination (see Mortimer et al Genome Biology and Evolution 2014).

Line 368-369 - "belies" is used incorrectly here. I think the authors meant that the phylogeographic uncertainty regarding the geographic origin of L4 is likely reflective of close relationships between ancestral strains in Europe and Africa. The term "belies" suggests that this impression is false.

The discussion of this phylogeographic uncertainty could be improved. The authors ask why some sub-lineages are observed only in Africa if L4 diversity is driven by colonial migrations to Africa. Some studies infer a European origin of L4 (Brynildsrud et al) whereas others infer an African origin (O'Neill). This phylogeographic uncertainty, as well as the observation of Africa-restricted sub-lineages of L4 suggests that the migratory history of L4 is more complicated than a model in which L4 was delivered exclusively via colonial European migrations to the continent. Rather, it seems plausible that bidirectional migrations occurred and as such, there is no real mystery in the differing geographic distributions of L4 sub-lineages.

Line 393-4 - "multiple studies" is repeated

**Reviewer 2**

I have now re-read the ms and the responses to reviewers comments. I'm glad to see that the authors took both sets of comments (I really like reviewer 1's comments) seriously. That has however undermined the strength and novelty of the manuscript. For example the major points of interest listed in the cover letter, namely - that a) Calcified nodules provide an unparalleled preservation environment for ancient DNA, b) Metagenomic screening of ancient DNA can identify past infections that gave rise to calcified nodules and c) A Bayesian dating analysis with a birth-death tree prior provides confirmation for the young age of the Mycobacterium tuberculosis complex (MTBC) - are not novel and don't change our understanding of TB evolutionary history.

I think the authors have done a very good job at attempting to address the bulk of the issues that myself and the other review reported on. And while I believe the veracity of the data collection to be second to none- I feel the clear variation in date estimates, the collapse of the estimates of R and the lack of resolution on lineage presence and directionality of movement, coupled with a discussion that largely re-iterates previously published work (see Oneill et al (https://onlinelibrary.wiley.com/doi/full/10.1111/mec.15120) - make the paper interesting but not novel.

In summary, I think the data are solid and there is nothing fundamentally wrong with the paper, just nothing particularly novel now that the corrections have been made.

**Authors Response**

**Point-by-point responses to the reviewers’ comments:**

Reviewer #1: The paper has been substantially improved in response to reviewer comments.

A few minor issues:

Line 209-210 - the authors state that LGT occurs infrequently among M. canettii. This is incorrect. Experimental conjugation is infrequently observed in laboratory settings (per Boritsch, which is cited here). Natural populations show evidence of frequent recombination (see Mortimer et al Genome Biology and Evolution 2014).

*We thank Reviewer 1 for pointing this out. We have removed “low frequency” from this sentence and have added Mortimer et al. 2014 as a citation for recombination in Mycobacterium canettii (see line 208 - 209).*

Line 368-369 - "belies" is used incorrectly here. I think the authors meant that the phylogeographic uncertainty regarding the geographic origin of L4 is likely reflective of close relationships between ancestral strains in Europe and Africa. The term "belies" suggests that this impression is false.

*We agree that the term is used incorrectly and have replaced it with “indicates” (line 366).*

The discussion of this phylogeographic uncertainty could be improved. The authors ask why some sub-lineages are observed only in Africa if L4 diversity is driven by colonial migrations to Africa. Some studies infer a European origin of L4 (Brynildsrud et al) whereas others infer an African origin (O'Neill). This phylogeographic uncertainty, as well as the observation of Africa-restricted sub-lineages of L4 suggests that the migratory history of L4 is more complicated than a model in which L4 was delivered exclusively via colonial European migrations to the continent. Rather, it seems plausible that bidirectional migrations occurred and as such, there is no real mystery in the differing geographic distributions of L4 sub-lineages.

*We do not disagree that the spread of Lineage 4 was likely driven by complex regional exchanges between Europe and Africa, and have now changed our phrasing at line 381. However, we believe that this still raises the question of why there is any geographical restriction of sublineages within Lineage 4.*

Line 393-4 - "multiple studies" is repeated

*We thank the reviewer for spotting this mistake and have edited the sentence at line 391.*

Reviewer #2: I have now re-read the ms and the responses to reviewers comments. I'm glad to see that the authors took both sets of comments (I really like reviewer 1's comments) seriously. That has however undermined the strength and novelty of the manuscript. For example the major points of interest listed in the cover letter, namely - that a) Calcified nodules provide an unparalleled preservation environment for ancient DNA, b) Metagenomic screening of ancient DNA can identify past infections that gave rise to calcified nodules and c) A Bayesian dating analysis with a birth-death tree prior provides confirmation for the young age of the Mycobacterium tuberculosis complex (MTBC) - are not novel and don't change our understanding of TB evolutionary history.

I think the authors have done a very good job at attempting to address the bulk of the issues that myself and the other review reported on. And while I believe the veracity of the data collection to be second to none- I feel the clear variation in date estimates, the collapse of the estimates of R and the lack of resolution on lineage presence and directionality of movement, coupled with a discussion that largely re-iterates previously published work (see Oneill et al (https://onlinelibrary.wiley.com/doi/full/10.1111/mec.15120) - make the paper interesting but not novel.

In summary, I think the data are solid and there is nothing fundamentally wrong with the paper, just nothing particularly novel now that the corrections have been made.

*Please see our letter to the editor requesting a re-visitation of the novelty question for our response to Reviewer 2’s comments.*
